# A High‐Entropy Oxyhydroxide with a Graded Metal Network Structure for Efficient and Robust Alkaline Overall Water Splitting

**DOI:** 10.1002/advs.202406008

**Published:** 2024-08-13

**Authors:** Chen‐Xu Zhang, Di Yin, Yu‐Xuan Zhang, Yu‐Xiang Sun, Xiao‐Jin Zhao, Wu‐Gang Liao, Johnny C. Ho

**Affiliations:** ^1^ State Key Laboratory of Radio Frequency Heterogeneous Integration (Shenzhen University) College of Electronics and Information Engineering Shenzhen 518060 China; ^2^ Department of Materials Science and Engineering City University of Hong Kong Hong Kong SAR 999077 P. R. China; ^3^ State Key Laboratory of Terahertz and Millimeter Waves City University of Hong Kong Hong Kong SAR 999077 P. R. China; ^4^ Institute for Materials Chemistry and Engineering Kyushu University Fukuoka 816‐8580 Japan

**Keywords:** graded metal network, heterostructure, high‐entropy oxyhydroxide, long‐term stability, water splitting

## Abstract

Designing high‐entropy oxyhydroxides (HEOs) electrocatalysts with controlled nanostructures is vital for efficient and stable water‐splitting electrocatalysts. Herein, a novel HEOs material (FeCoNiWCuOOH@Cu) containing five non‐noble metal elements derived by electrodeposition on a 3D double‐continuous porous Cu support is created. This support, prepared via the liquid metal dealloying method, offers a high specific surface area and rapid mass/charge transfer channels. The resulting high‐entropy FeCoNiWCuOOH nanosheets provide a dense distribution of active sites. The heterostructure between Cu skeletons and FeCoNiWCuOOH nanosheets enhances mass transfer, electronic structure coupling, and overall structural stability, leading to excellent activities in the oxygen evolution reaction (OER), hydrogen evolution reaction (HER), and water splitting reaction. At 10 mA cm^−2^, the overpotentials for OER, HER, and water splitting in 1.0 m KOH solution are 200, 18, and 1.40 V, respectively, outperforming most current electrocatalysts. The catalytic performance remains stable even after operating at 300 mA cm^−2^ for 100, 100, and over 1000 h, correspondingly. This material has potential applications in integrated hydrogen energy systems. More importantly, density functional theory (DFT) calculations demonstrate the synergy of the five elements in enhancing water‐splitting activity. This work offers valuable insights for designing industrial water electrolysis systems.

## Graphical abstract

A 3D double continuous porous structure with a high entropy layer of double hydroxide is synthesized. The Cu carrier endows a high specific surface area, and the derived high‐entropy FeCoNiWCuOOH nanosheets provide high‐density and widely distributed active sites. Ultimately, efficient water‐splitting for hydrogen production is achieved by various green energy sources.

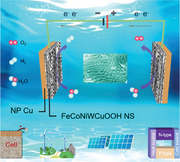

## 1 Introduction

Hydrogen energy is one of the most promising ways to achieve decarbonization goals, owing to its sustainability, high energy density, and environmentally friendly attributes. Electrocatalytic water splitting has become an ideal strategy for converting intermittent renewable energy into hydrogen fuel.^[^ ^1^, ^2^, ^3^, ^4^ ^]^ Despite the current prominence of catalysts containing precious metals like Pt and Ir for this process, their scarcity and high cost severely constrain their widespread practical applications. Consequently, extensive research efforts are directed toward developing low‐cost and efficient electrocatalysts based on transition metals abundant in the Earth's crust.^[^ ^5^, ^6^, ^7^ ^]^ Single transition metals (Fe, Co, Ni, W, Mo, Cu, etc.) typically exhibit moderate catalytic activity, whereas alloys comprising these elements can modulate the number of active site moles (geometric effects) and electronic structures (electronic effects),^[^ ^8^, ^9^, ^10^, ^11^, ^12^, ^13^ ^]^ thereby generating an ideal catalytic transition state with the minimum energy barrier, continuously improving the performance of electrocatalytic water splitting.

HEOs are a class of emerging materials composed of five or more primary metallic elements.^[^ ^14^, ^15^, ^16^ ^]^ They consist of oxyhydroxides and high‐entropy structures. Oxyhydroxides exhibit diversity in elemental composition, flexibility in structure, and simplicity in preparation, making them one of the most effective electrocatalysts for water splitting.^[^ ^17^, ^18^ ^]^ High‐entropy engineering holds promise for further enhancing the electrocatalytic water‐splitting performance of oxyhydroxides.^[^ ^19^, ^20^ ^]^ Through the synergistic effect of multiple metal components, precise control of geometric morphology, optimized electronic structure, and outstanding chemical and thermal stability,^[^ ^21^, ^22^, ^23^ ^]^ HEOs demonstrate enormous potential in energy storage and electrocatalysis.

In addition to selecting electrocatalytic water‐splitting materials with high intrinsic activity, facilitating the adsorption, reaction, and desorption of reactants with a large specific surface area is crucial for effectively harnessing the high catalytic activity of high‐entropy materials.^[^ ^24^, ^25^, ^26^ ^]^ Apart from widely studied particle catalysts, nanoporous metals, serving as structure‐function integrated materials, possess a 3D double‐continuous open porous structure, high specific surface area, and high conductivity, which promote mass transfer within internal porous channels.^[^ ^27^, ^28^, ^29^ ^]^ Moreover, nanoporous matrices readily integrate with other active substances, enabling template strategies to precisely control the loading amount and interface structure, further enhancing catalytic performance.^[^ ^30^ ^]^

Herein, we have developed a novel FeCoNiWCuOOH@Cu heterostructure HEOs material, leveraging the structural advantages of 3D double‐continuous pores and the dual‐component design concept of HEOs, exhibiting high catalytic activity and long‐term stability for efficient electrocatalytic alkaline water splitting reactions. The optimized FeCoNiWCuOOH@Cu electrode demonstrated an excellent performance in the OER/HER/water splitting reactions with overpotentials of 200, 18, and 1.40 V, respectively, at 10 mA cm^−2^ in KOH (1 m) solution. Notably, at a high current density of 300 mA cm^−2^, it exhibited remarkable durability and stability, operating for 100, 100, and 1000 h or more. Among them, the Cu carrier, prepared using the liquid dealloying method, features a 3D double‐continuous porous structure, providing advantages such as high conductivity, high specific surface area, and rapid charge/mass transfer. By leveraging the structural advantages of Cu carriers, FeCoNiWCuOOH NS growth was sufficiently promoted, ensuring the enrichment and widespread distribution of multiple active sites. The high‐entropy “cocktail” effect, arising from the multi‐element composition and unique microstructure of HEOs, led to unexpected nonlinear results. The entropy stabilization effect near the surface effectively countered the influence of surface electrochemical oxidation‐reduction, resulting in long‐term stable activity. Further, DFT calculations demonstrated that the synergistic effect of the five elements enhanced the electrocatalytic water‐splitting activity of the catalyst. The rich selectivity of elements and precise regulation of nanostructures injected new vitality into the industrial application of high‐entropy catalysts in long‐term and efficient electrocatalytic water‐splitting reactions.

## 2 Results and Discussion

### 2.1 Synthesis and Structural Characterization of FeCoNiWCuOOH@Cu System Electrocatalysts

**Figure 1:**
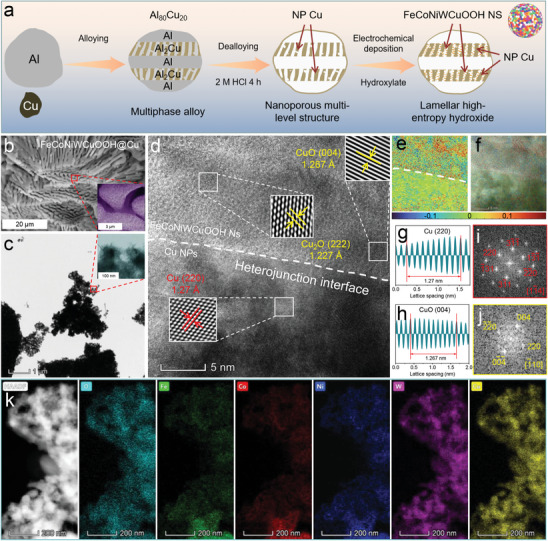
Microstructure properties of FeCoNiWCuOOH@Cu. a) Synthesis diagram, b) SEM, and c) TEM images, the inset of (b) and (c) are the enlarged view of the corresponding positions, respectively, d) HRTEM image, e,f) strain distribution along *E*_xy_ of (d), the compressive strain showed a shift from green to dark blue, and the tensile strain showed a shift from bright yellow to red, g,h) the measured interplanar distances, i,j) FFT patterns, and k) HAADF‐STEM image and its corresponding EDS elemental mappings of FeCoNiWCuOOH@Cu.

The FeCoNiWCuOOH@Cu catalyst was synthesized using a simple and scalable alloy‐dealloying method combined with an electrodeposition technique (see Experimental Section, **Figure** **1**a; Figure S1, Supporting Information, for details). The preparation of AlCu alloy was achieved through sufficient mixing of liquid metals using the high‐temperature alloying method. X‐ray diffraction (XRD), scanning electron microscope (SEM), and Brunauer‐Emmett‐Teller (BET) tests (Figure S2, Supporting Information) confirmed that the AlCu alloy is an intermetallic compound composed of Al and Al_2_Cu, featuring a dense structure without voids, resulting in a specific surface area of only 0.882 m^2^ g^−1^. Subsequently, upon chemical dealloying of Al element with 2 m hydrochloric acid solution, the intermetallic compound gradually transformed into a double‐continuous pore Cu framework with interconnected large channels and small nanopores. Notably, chloride ions played a crucial role in promoting the surface diffusion of Cu, facilitating the formation of larger ligaments, and enhancing anti‐coarsening stability.^[^ ^31^ ^]^ To further improve activity and stability, chemically active 3D transition metals such as Fe, Co, Ni, and 5d metal W were introduced into Cu. FeCoNiWCuO was in situ grown on the Cu framework using the electrochemical deposition method, and FeCoNiWCuOOH NS were generated through electrochemical oxidation. The double hydroxide layer on the surface played a vital role in enhancing anti‐coarsening stability by reducing surface energy.^[^ ^31^ ^]^ Cu, CuOOH@Cu, FeCuOOH@Cu, FeWCuOOH@Cu, and FeNiWCuOOH@Cu samples were prepared separately using the same method.

The SEM images of FeCoNiWCuOOH@Cu (Figure 1b) reveal a typical hierarchical porous structure consisting of interpenetrating large channels and small nanopores within interconnected Cu ligaments. From the enlarged view in Figure 1b, many nanoparticles (NPs) can be observed forming the skeleton of this continuous channel structure, filled with secondary nanoporous structures. These structures are beneficial for increasing the specific surface area, facilitating the widespread distribution of active sites, and promoting electrolyte diffusion, thereby enhancing effective electrochemical reactions on the catalyst surface.^[^ ^32^, ^33^ ^]^ In the transmission electron microscope (TEM) image (Figure 1c), the loose porous surface of the FeCoNiWCuOOH@Cu catalyst is observed to be filled with ultra‐thin NS structures, identified as FeCoNiWCuOOH NS formed through the electrochemical reduction process. This ultrathin NS structure effectively optimizes the diffusion distance and charge transfer rate between reactants and products, thereby accelerating mass/charge transfer during the catalytic process. Notably, the surface‐distributed small nanopores have an average size of ≈10 nm and are evenly distributed on the surface of the Cu framework.^[^ ^34^ ^]^ The type III nitrogen adsorption/desorption isotherm of the hierarchically porous FeCoNiWCuOOH@Cu catalyst exhibits accelerated adsorption characteristics. The nanopore size distribution shows a distinct peak centered at ≈11.85 nm, indicative of a typical nanopore structure formed by Cu NPs (Figure S3, Supporting Information).

High‐resolution TEM (HR‐TEM) was employed to investigate the detailed atomic structure of FeCoNiWCuOOH@Cu (Figure 1d). A distinct heterostructure between Cu NPs and FeCoNiWCuOOH NS is observed, indicating a continuous and stable heterostructure with strong coupling effects. This facilitates efficient charge transfer and further enhances the activity and stability of the system through synergistic effects.^[^ ^35^, ^36^ ^]^ Furthermore, the high‐temperature melting reaction produces short‐ and long‐range ordering of Cu ligaments. Due to the mismatch in element mass, size, and bond state, severe lattice distortion occurs, leading to short‐range disordered lattice stripes in the FeCoNiWCuOOH material. This is confirmed by the geometric phase analysis (GPA) image (Figure 1e) obtained from Figure 1d, further confirming the multi‐element solid solution structure of FeCoNiWCuOOH.^[^ ^37^ ^]^ Comparing HRTEM with GPA images (Figure 1f), it is evident that lattice strain primarily occurs in the FeCoNiWCuOOH structure, whereas lattice strain is less evident in the Cu skeleton. This difference is also a contributing factor to the high activity of FeCoNiWCuOOH. Additionally, the stability of the FeCoNiWCuOOH material's structure maintains orderly long‐range atom arrangements, thereby sustaining a stable charge transport structure. HR‐TEM images of Cu NPs and FeCoNiWCuOOH NS reveal lattice fringes with spacings of 1.27 (Figure 1d,g,i), 1.227, and 1.267 Å (Figure 1d,h,j), respectively, corresponding to the (220), (222), and (004) diffraction facets of Cu, Cu_2_O, and CuO. This indicates that FeCoNiWCuOOH NS grows on the Cu skeleton composed of Cu NPs, and the high‐entropy NS structure still mainly exhibits a copper oxide crystal structure. The atomic ratios of various elements in the FeCoNiWCuOOH@Cu catalyst (Fe/Co/Ni/W/Cu/O = 4.05/5.52/5.29/0.01/43.89/41.24) were analyzed using TEM‐related energy dispersive X‐ray spectroscopy (EDS) (Figure S4, Supporting Information). The high content of Cu and O indicates the generation of a certain amount of copper oxide in the system. Aberration‐corrected high‐angle annular dark‐field scanning transmission electron microscopy (HAADF‐STEM) image and related EDS elemental imaging distribution diagrams (Figure 1k) demonstrate a uniform distribution of Fe, Co, Ni, W, Cu, and O elements in the FeCoNiWCuOOH@Cu catalyst. Additionally, Analysis of the HRTEM and corresponding GPA images (Figure S5, Supporting Information) reveals that the formation of a hydroxide oxide layer on the Cu substrate surface enhances its resistance to coarsening. As the number of metal elements increases, the hydroxide oxide layer exhibits more kinks and step sites, leading to more grain boundaries and defects. This is indicative of the strain and cocktail effects. Moreover, SEM morphology, EDS element distribution, and atomic ratios of Cu (Figure S6, Supporting Information), CuOOH@Cu (Figure S7, Supporting Information), FeCuOOH@Cu (Figure S8, Supporting Information), FeWCuOOH@Cu (Figure S9, Supporting Information) and FeNiWCuOOH@Cu (Figure S10, Supporting Information) exhibit similar characteristics.

### 2.2 Phase Structure and Electronic State Analysis of FeCoNiWCuOOH@Cu System Electrocatalysts

**Figure 2:**
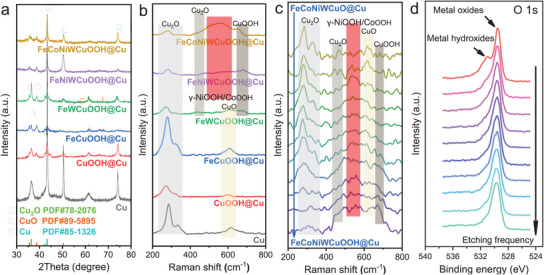
Chemical characterization. a) XRD patterns, b) Raman patterns, c) in situ Raman spectroscopy, and d) XPS spectra of O 1s of different samples.

The composition of the FeCoNiWCuOOH@Cu system was further characterized by X‐ray diffraction (XRD) patterns (**Figure** **2**a). The results reveal that the primary structure of the double‐continuous pore framework consists of Cu elements. However, the presence of Cu_2_O was observed due to the weak oxidation of the Cu framework by oxygen in both air and solution. Following the electrochemical hydroxylation process, Cu_2_O gradually oxidizes to CuO, as evidenced by the appearance of CuO diffraction peaks in the XRD curves of CuOOH@Cu, FeCuOOH@Cu, FeWCuOOH@Cu, FeNiWCuOOH@Cu, and FeCoNiWCuOOH@Cu. Moreover, due to the amorphous nature of the hydroxyl oxide formed on the surface of the Cu skeleton, no corresponding diffraction peaks are observed in the XRD pattern. Hence, Raman spectroscopy testing (Figure 2b) was conducted to detect the presence of hydroxyl oxides. The results indicate the detection of not only CuO and Cu_2_O after electrochemical hydroxylation but also the presence of CuOOH and γ‐NiOOH/CoOOH.^[^ ^38^, ^39^, ^40^, ^41^ ^]^ To monitor real‐time changes in crystal structure during the electrochemical hydroxylation process, in situ electrochemical Raman spectroscopy testing was performed on FeCoNiWCuOOH@Cu (Figure 2c; Figure S11, Supporting Information). The findings reveal a gradual weakening of the Cu_2_O peak and an increase in the CuO peak as the electrochemical hydroxylation process progresses, suggesting the progressive oxidation of Cu_2_O to CuO. Additionally, the peak corresponding to CuOOH and γ‐NiOOH/CoOOH increases gradually during the electrochemical process, indicating the gradual formation of an amorphous metal hydroxyl oxide layer on the surface of the Cu skeleton. In the high wavenumber range of the Raman plot (Figure S12, Supporting Information), no peaks corresponding to common intercalated anions (e.g., NO_3_^−^,^[^ ^42^ ^]^ CO_3_^2‐^.^[^ ^43^ ^]^) were observed. This confirms that the generated structure is a Metal OOH rather than a layered double hydroxide. Inert gas ion bombardment was utilized to sequentially etch the surface of the FeCoNiWCuOOH@Cu sample layer by layer, and the X‐ray photoelectron spectroscopy (XPS) spectra of O 1s after each etching step were examined (Figure 2d). The results indicate a gradual disappearance of the metal hydroxide peak with increasing etching depth, while the metal oxide peak still exists. This further confirms the transformation of the metal oxide layer on the sample's surface into a metal oxide/metal hydroxyl oxide layer during the electrochemical hydroxylation process. Based on comprehensive XRD, Raman, in−situ electrochemical Raman, and XPS analyses, it can be concluded that FeCoNiWCuO gradually transforms into FeCoNiWCuOOH during the electrochemical hydroxylation process, rendering it suitable for subsequent electrocatalytic testing.

XPS was utilized to investigate the near‐surface electronic structure and oxidation state of all samples. The XPS wide scan measurement spectrum confirmed the presence of Fe, Co, Ni, W, Cu, and O elements (Figure S13, Supporting Information). High‐resolution XPS spectra (Figures S14–S19, Supporting Information) of corresponding O 1s, Cu 2p, Fe 2p, W 4f, Ni 2p, and Co 2p for Cu, CuOOH@Cu, FeCuOOH@Cu, FeWCuOOH@Cu, FeNiWCuOOH@Cu, and FeCoNiWCuOOH@Cu were collected. The O 1s spectra consist of metal oxide peaks attributed to Cu_2_O/CuO and metal hydroxide peaks attributed to Cu(OH)_2_. Cu 2p spectra can be indexed as the Cu^0^ peak of the Cu skeleton, the Cu^1+^ peak of Cu_2_O, and the Cu^2+^ peak of CuO. Fe 2p spectra can be deconvoluted into two pairs of Fe^2+^ and Fe^3+^, along with a pair of satellite peaks. The W 4f spectra can be attributed to W^6+^, while Ni 2p spectra can be indexed as the Ni^2+^ peak and a pair of satellite peaks. Co 2p spectra can be deconvoluted into two pairs of Co^2+^ and Co^3+^, along with a pair of satellite peaks. Due to the influence of electrochemical deposition and hydroxylation reactions, all metal elements in FeCoNiWCuOOH@Cu mainly exist in high oxidation states, which is beneficial for alkaline OER to exhibit high electrocatalytic activity. Additionally, oxygen‐related species in FeCoNiWCuOOH@Cu are often considered active sites for catalyzing OER.^[^ ^44^ ^]^ Comparing the XPS fine spectra of Cu 2p before and after the hydroxylation process (Figure S20, Supporting Information), it can be seen that after electrochemical hydroxylation, the Cu^2+^ peak of Cu in XPS shifts to a higher energy level, indicating the formation of CuOOH.^[^ ^45^ ^]^

### 2.3 OER Activity of FeCoNiWCuOOH@Cu System Electrocatalysts

**Figure 3:**
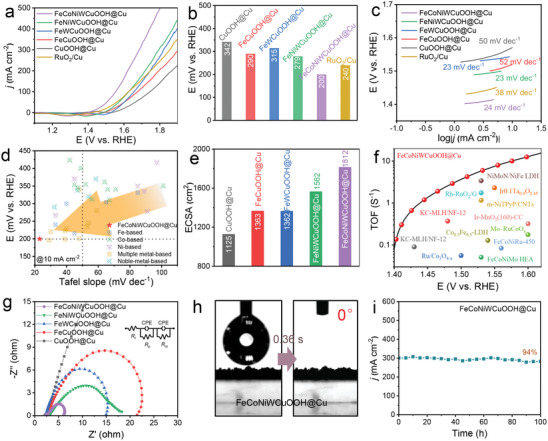
Electrochemical characterization on OER properties. a) Polarization curves, b) corresponding overpotentials, and c) Tafel slopes of different samples in 1 m KOH solution. d) Overpotentials at 10 mA cm^−2^ of the as‐obtained FeCoNiWCuOOH@Cu electrocatalyst in comparison with reported related electrocatalysts. e) ECSA histograms of different samples. f) TOF pattern of the as‐obtained FeCoNiWCuOOH@Cu electrocatalyst in contrast with reported related electrocatalysts. g) EIS curves of other samples. h) Contact angle photos of FeCoNiWCuOOH@Cu. i) Constant current curve of FeCoNiWCuOOH@Cu at 300 mA cm^−2^ current density for 100 h.

The electrocatalytic activity for alkaline OER of FeCoNiWCuOOH@Cu system catalysts in 1 m KOH electrolyte was evaluated using linear sweep voltammetry (LSV) in a three‐electrode system, with commercial RuO_2_/Cu catalysts measured and studied for comparison. From the polarization curves depicted in **Figure** **3**a,b, it is evident that the FeCoNiWCuOOH@Cu sample exhibits superior OER performance. At a current density of 10 mA cm^−2^, FeCoNiWCuOOH@Cu requires an overpotential of only 200 mV, which is notably better than FeNiWCuOOH@Cu (279 mV), FeWCuOOH@Cu (315 mV), FeCuOOH@Cu (290 mV), CuOOH@Cu (341 mV), and RuO_2_/Cu (240 mV) catalysts. The corresponding Tafel curves (Figure 3c) further indicate that FeCoNiWCuOOH@Cu possesses a lower Tafel slope of 24 mV dec^−1^, significantly smaller than FeNiWCuOOH@Cu (23 mV dec^−1^), FeWCuOOH@Cu (23 mV dec^−1^), FeCuOOH@Cu (52 mV dec^−1^), CuOOH@Cu (50 mV dec^−1^), and RuO_2_/Cu (38 mV dec^−1^) catalysts. This highlights the rapid OER kinetic charge transfer of FeCoNiWCuOOH@Cu.^[^ ^46^ ^]^ Furthermore, the double‐continuous pore structure of the FeCoNiWCuOOH@Cu catalyst not only utilizes industrially scalable preparation methods (produced using industrial‐scale metallurgical techniques (ranging from tons to millions of tons) but also employs simple dealloying and electrochemical deposition processes to achieve OER activity). Its performance significantly surpasses other noble metal and non‐noble metal‐based electrocatalysts (Figure 3d; Table S1, Supporting Information), demonstrating its enormous potential for near‐term commercialization.^[^ ^47^ ^]^ In addition, we conducted the electrochemical tests of the catalysts before and after the electrochemical activation process (hydroxylation) (Figure S21, Supporting Information). The results showed that due to the introduction of high‐entropy structures, the active sites of the catalysts gradually increased (lattice distortion led to many kinks, step edges, and grain boundaries), resulting in a gradual improvement in electrochemical performance.

Subsequently, the OER electrochemical active surface area (ECSA) of the catalyst was measured using double‐layer capacitance (C_dl_) to gain a deeper understanding of the intrinsic catalytic activity of the FeCoNiWCuOOH@Cu system. The C_dl_ is derived from the cyclic voltammetry (CV) curve (Figure S22, Supporting Information). According to calculations, the ECSA value of FeCoNiWCuOOH@Cu can reach 1812 cm^2^ (Figure 3e), ≈1.61, 1.32, 1.33, and 1.16 times that of CuOOH@Cu, FeCuOOH@Cu, FeWCuOOH@Cu, and FeNiWCuOOH@Cu, respectively. The large ECSA value of FeCoNiWCuOOH@Cu indicates that owing to the porous double‐continuous pore structure and various metal components, it not only provides a sizeable electrochemical interface and more active sites but also enhances the availability of active sites in electrocatalytic reactions and promotes the adsorption of water molecules and close contact with electrolytes. The intrinsic activity of FeCoNiWCuOOH@Cu was evaluated using the ECSA normalized LSV curves, as shown in Figure S23 (Supporting Information). FeCoNiWCuOOH@Cu exhibits significant intrinsic activity, requiring only 256 mV of overpotential to achieve a normalized current density of 0.01 mA cm^−2^ for ECSA. This value is significantly lower than CuOOH@Cu (377 mV), FeCuOOH@Cu (331 mV), FeWCuOOH@Cu (342 mV), and FeNiWCuOOH@Cu (316 mV). The results indicate that the high‐entropy structure, heterostructure, and double‐continuous pore structure can all improve catalytic performance, with the first two enhancing intrinsic catalytic activity and the latter significantly increasing available active sites.^[^ ^48^, ^49^, ^50^ ^]^ To further evaluate the intrinsic activity of the catalyst, the O_2_ turnover frequency (TOF) of FeCoNiWCuOOH@Cu was calculated (Figure S24, Supporting Information) and compared with recently reported related catalysts (Figure 3f; Table S2, Supporting Information). The results show that FeCoNiWCuOOH@Cu has excellent TOF values. At overpotentials of 200 and 300 mV, the TOF values are 0.764 and 5.806 s^−1^, respectively, better than those of the recently reported related catalysts. In addition, more evaluations were conducted using mass activity analysis. The results indicate that the mass activity of FeCoNiWCuOOH@Cu is the highest, reaching 150.08 A g^−1^ at 300 mV (Figure S25, Supporting Information).

The Nyquist plots obtained through electrochemical impedance spectroscopy (EIS) illustrate the corresponding charge transfer resistance (*R*_ct_) semicircles, thereby investigating the electron transfer kinetics at the catalyst/electrolyte interface. As depicted in Figure 3g, the EIS spectrum of the nanoporous FeCoNiWCuOOH@Cu electrode displays two characteristic semicircles in the mid to low‐frequency range, aligned with the constant phase element (CPE), representing *R*_ct_ and pore resistance (*R*_p_), respectively.^[^ ^51^, ^52^ ^]^ At higher frequencies, the real axis intercept denotes the intrinsic resistance (*R*_I_) of the electrode and electrolyte. Based on the equivalent circuit of these universal descriptors (illustrated in Figure 3g), the *R*_ct_ value of nanoporous FeCoNiWCuOOH@Cu is determined to be ≈3.7 Ω, significantly lower than that of nanoporous CuOOH@Cu (≈94.29 Ω), FeCuOOH@Cu (≈22.48 Ω), FeWCuOOH@Cu (≈14.36 Ω), and FeNiWCuOOH@Cu (≈13.31 Ω) (Figure S26, Supporting Information). The *R*_I_ and *R*_p_ values of FeCoNiWCuOOH@Cu are ≈2.19 and 0.97 Ω, respectively, comparable to those of nanoporous CuOOH@Cu (≈2.06 and 0.79 Ω), FeCuOOH@Cu (≈2.78 and 4.89 × 10^−4^ Ω), FeWCuOOH@Cu (≈2.21 and 0.14 Ω), and FeNiWCuOOH@Cu (≈3.13 and 0.97 Ω). This suggests that the high‐entropy structure has minimal impact on the intrinsic resistance and pore resistance of the system, possibly due to the stable heterostructure formed between the Cu skeleton and the high‐entropy structure. Consequently, the entire system demonstrates the lowest responsive charge transfer resistance and faster electron transfer process, consistent with its excellent OER catalytic performance. Further insights into the adsorption strength of the catalyst for water molecules were obtained through contact angle testing. As depicted in Figure 3h, the nanoporous FeCoNiWCuOOH@Cu catalyst achieves nearly instantaneous (0.36 s) overall infiltration and adsorption of water molecules (≈0°), indicating the loose and porous characteristics of the double‐continuous pore structure and the high adsorption capacity of the high‐entropy structure for water molecules, thereby facilitating the adsorption of water molecules and subsequent decomposition reactions. Moreover, nanoporous CuOOH@Cu, FeCuOOH@Cu, FeWCuOOH@Cu, and FeNiWCuOOH@Cu also exhibit excellent hydrophilicity (Figure S27, Supporting Information), highlighting the unique advantage of the sample preparation method in the electrocatalytic water splitting reaction.

Durability stands as a key indicator in designing practical OER electrocatalysts. The durability of FeCoNiWCuOOH@Cu was assessed through extended timed current testing (Figure 3i). Under the alkaline condition with a fixed current density of 300 mA cm^−2^, following a prolonged 100 h OER reaction, the current density of FeCoNiWCuOOH@Cu merely decreased by 6%. This outcome indicates the excellent stability of the FeCoNiWCuOOH@Cu electrode. To investigate changes in the FeCoNiWCuOOH@Cu crystal structure pre‐ and post‐stability experiments, XRD and Raman spectroscopy analyses were conducted. As depicted in Figure S28a (Supporting Information), the persistence of both the Cu skeleton and its surface oxides is evident. Post‐stability testing shows a gradual increase in the peak intensity of the oxides, indicating further enhancement in surface Cu oxidation. This is further corroborated by the enhanced CuOOH peak in the Raman spectrum (Figure S28b, Supporting Information). Moreover, diverse elements and diffraction peaks in the XPS spectrum (Figure S29, Supporting Information) imply that post‐stability experiments, the crystal structure and elemental valence states of FeCoNiWCuOOH@Cu have undergone negligible changes. Furthermore, to study morphological changes in FeCoNiWCuOOH@Cu, SEM images were examined post‐stability testing. Figure S30 (Supporting Information) illustrates that the nanoporous double‐continuous pore structure remains intact, with a relatively uniform distribution of various elements. This may be attributed to the entropy stabilization effect of the high‐entropy surface, which prevents electrochemical redox near the surface. At the same time, FeCoNiWCuOOH@Cu heterostructure stabilizes the connection, ensuring system stability. These findings indicate the remarkable structural stability of FeCoNiWCuOOH@Cu during prolonged electrochemical OER processes, demonstrating its potential as an efficient OER catalyst.

### 2.4 HER Activity of FeCoNiWCuOOH@Cu System Electrocatalysts

**Figure 4:**
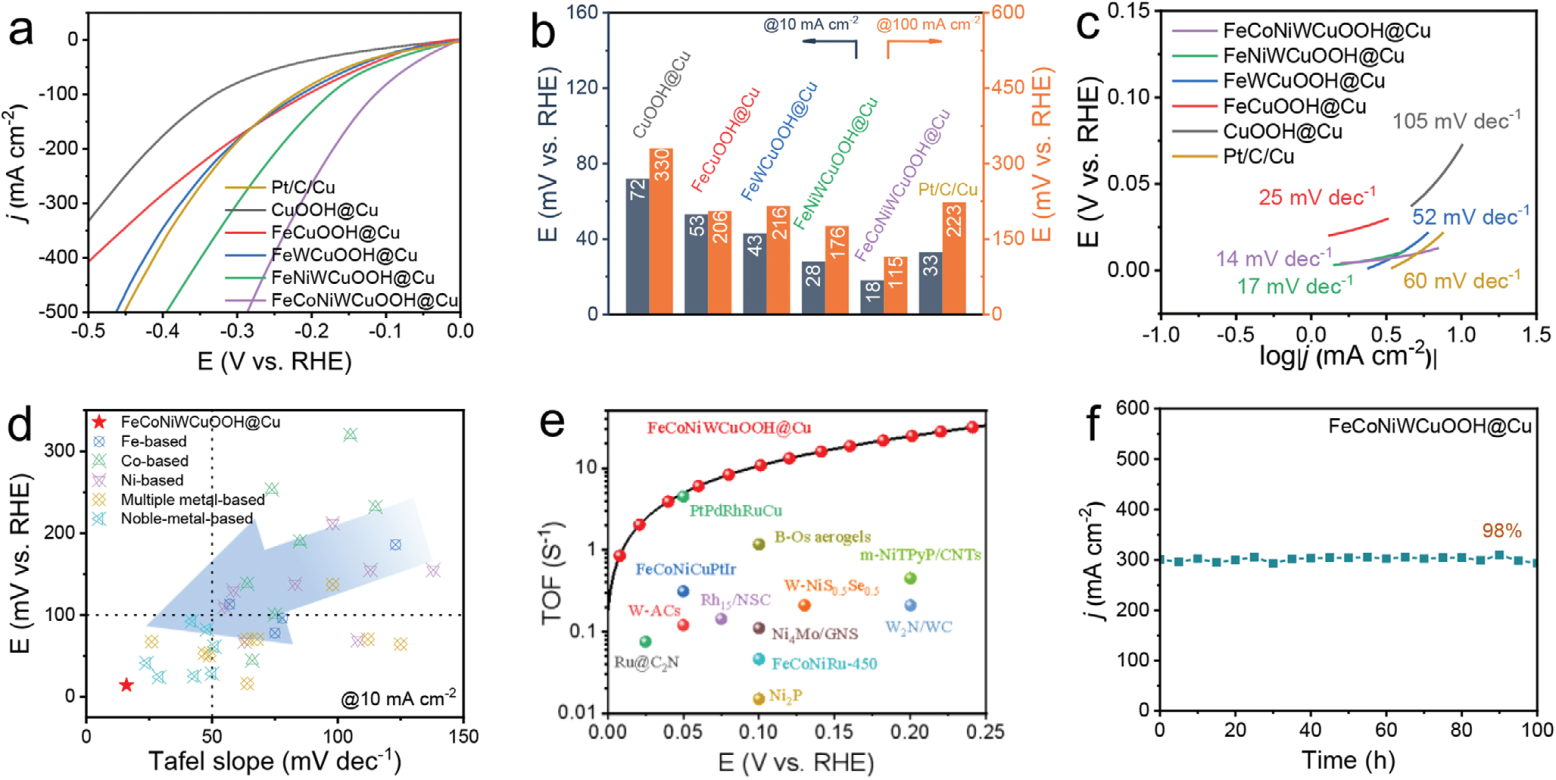
Electrochemical characterization on HER properties. a) Polarization curves, b) corresponding overpotentials, c) Tafel slopes of different samples in 1 m KOH solution. d) Overpotentials at 10 mA cm^−2^ and e) TOF pattern of the as‐obtained FeCoNiWCuOOH@Cu electrocatalyst compared with reported related electrocatalysts. f) Constant current curve of FeCoNiWCuOOH@Cu at 300 mA cm^−2^ current density for 100 h.

The HER performance of FeCoNiWCuOOH@Cu system catalysts featuring a double‐continuous pore structure was investigated under alkaline conditions. Analysis of the polarization curves depicted in **Figure** **4**a reveals that compared to CuOOH@Cu (72 mV), FeCuOOH@Cu (53 mV), FeWCuOOH@Cu (43 mV), and FeNiWCuOOH@Cu (28 mV), the FeCoNiWCuOOH@Cu catalyst exhibits the lowest overpotential of 18 mV, enabling a current density of 10 mA cm^−2^, which is notably lower than the 33 mV overpotential of commercial Pt/C/Cu (Figure 4b). Remarkably, the FeCoNiWCuOOH@Cu catalyst achieves a substantial overpotential of 115 mV at 100 mA cm^−2^, significantly lower than CuOOH@Cu (330 mV), FeCuOOH@Cu (206 mV), FeWCuOOH@Cu (216 mV), FeNiWCuOOH@Cu (176 mV), and Pt/C/Cu (223 mV). To further delve into the HER reaction kinetics, the Tafel slope was derived from the corresponding polarization curves. As illustrated in Figure 4c, the Tafel slope value provided by FeCoNiWCuOOH@Cu stands at 14 mV dec^−1^, substantially smaller than that of CuOOH@Cu (105 mV dec^−1^), FeCuOOH@Cu (25 mV dec^−1^), FeWCuOOH@Cu (52 mV dec^−1^), FeNiWCuOOH@Cu (17 mV dec^−1^), and Pt/C/Cu (60 mV dec^−1^). This suggests that the rapid HER kinetics of FeCoNiWCuOOH@Cu are rooted in the Volmer–Tafel mechanism, dominated by the Tafel step under the alkaline condition. Additionally, by comparing the electrochemical test results before and after the electrochemical activation process (hydroxylation) (Figure S31, Supporting Information), we found that the catalyst activity is further enhanced after hydroxylation. Metal‐hydroxide oxide (M‐OOH) can further improve water electrolysis activity. To elucidate the outstanding performance achieved by the nanoporous double‐continuous pore structure of FeCoNiWCuOOH@Cu, a comparison was made with numerous recently reported excellent HER electrocatalysts (Figure 4d; Table S3, Supporting Information), including Fe, Co, Ni, multiple metals, and noble metal‐based materials. It was observed that the FeCoNiWCuOOH@Cu catalyst exhibits excellent HER activity with a lower Tafel slope. Additionally, the H_2_ TOF of FeCoNiWCuOOH@Cu was assessed to further elucidate its intrinsic activity. The results (Figure 4e; Table S4, Supporting Information) demonstrate that at overpotentials of 50, 100, and 200 mV, the TOF values stand at 5.166, 12.766, and 41.292 s^−1^, respectively. These values surpass those of most recently reported related catalysts, indicating that high‐entropy structures enhance intrinsic activity. Consistent with the HER polarization curve results, the FeCoNiWCuOOH@Cu catalyst displayed the highest mass activity value (55.77 A g^−1^ at −50 mV) in the samples (Figure S32, Supporting Information). This means that the excellent HER performance of FeCoNiWCuOOH@Cu under alkaline conditions may be attributed to the synergistic effect of multiple nonprecious metal components.

As is well known, exceptional electrocatalysts typically exhibit sustained durability over prolonged periods or under high current densities. The stability of the catalyst was assessed via chronoamperometry, as depicted in Figure 4f. At a high current density of 300 mA cm^−2^, FeCoNiWCuOOH@Cu demonstrates the ability to maintain a stable current output for a minimum of 100 h, with a current density of 98% and almost no activity decay, demonstrating good durability. The crystal structure and morphology changes of the FeCoNiWCuOOH@Cu catalyst post‐durability testing were evaluated using XRD, Raman, and SEM techniques. XRD patterns of the FeCoNiWCuOOH@Cu catalyst pre‐ and post‐durability testing were analyzed (Figure S33a, Supporting Information). A comparison reveals that following the 100‐h stability test, the peak intensity of CuO gradually weakens while that of Cu_2_O strengthens. This phenomenon is attributed to the reduction reaction occurring at the cathode, leading to the gradual transition of Cu from a high valence state to a low valence state. This transition is further evident in the weakened Cu_2_O and γ‐NiOOH/CoOOH peaks observed in the Raman spectrum (Figure S33b, Supporting Information). SEM analysis (Figure S34, Supporting Information) reveals that following the 100‐h durability test, the FeCoNiWCuOOH@Cu catalyst retains its nanoporous double‐continuous pore structure composed of nanoparticles, with no significant changes observed in the corresponding element mappings and EDS spectra. This indicates the excellent structural stability of the FeCoNiWCuOOH@Cu catalyst, with changes post‐long‐term durability testing deemed negligible.

### 2.5 Water Splitting Activity of FeCoNiWCuOOH@Cu System Electrocatalysts

**Figure 5:**
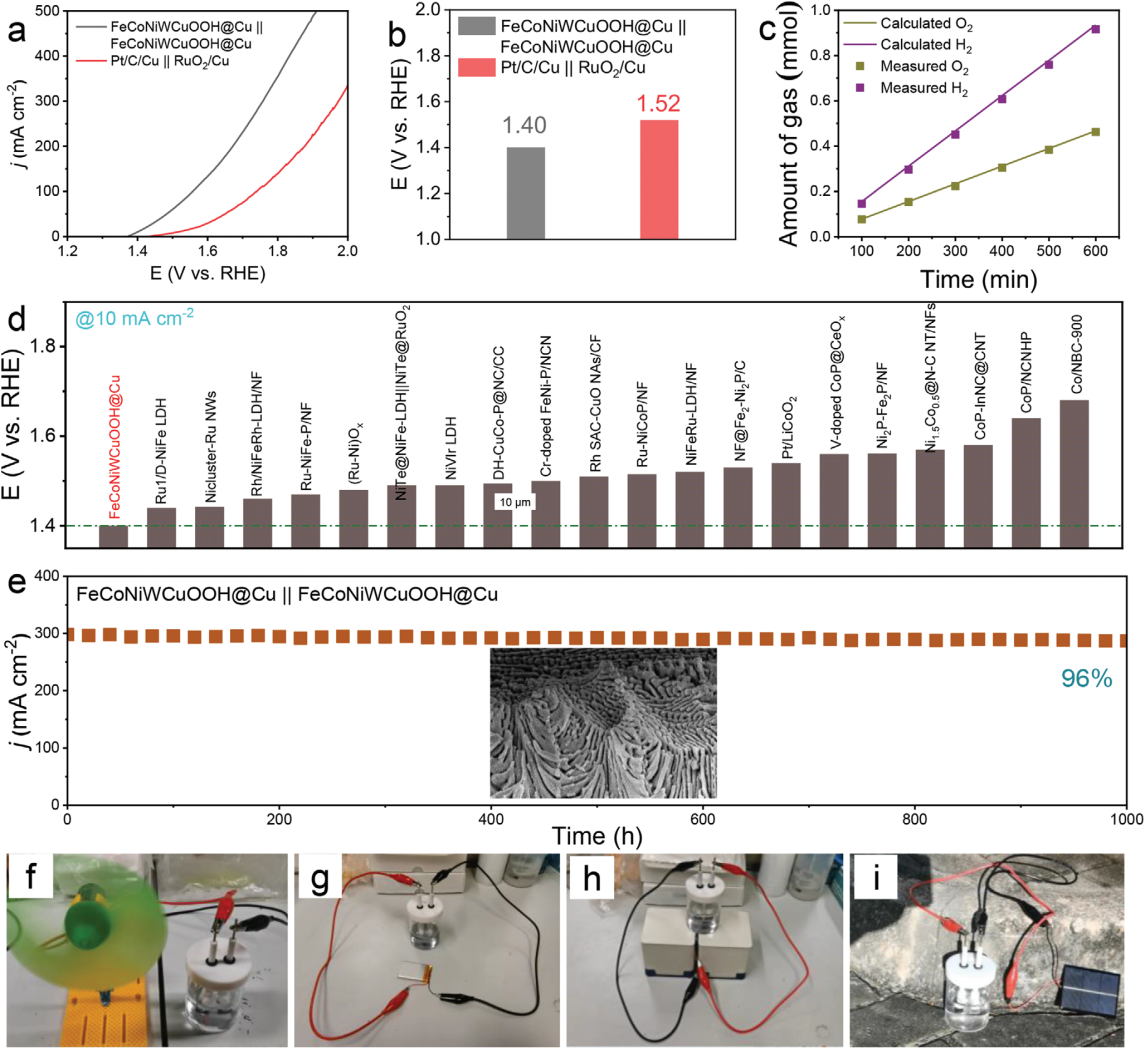
Electrochemical performance in alkaline water electrolyzer. a) Polarization curves and b) corresponding overpotentials at 10 mA cm^−2^ of different samples in 1 m KOH aqueous electrolyte. c) FE of H_2_ and O_2_ generated over FeCoNiWCuOOH@Cu electrode at 10 mA cm^−2^ for 600 min. d) Overpotentials of the as‐obtained FeCoNiWCuOOH@Cu electrocatalyst at 10 mA cm^−2^ compared to reported related electrocatalysts. e) Constant current curve of FeCoNiWCuOOH@Cu||FeCoNiWCuOOH@Cu at 300 mA cm^−2^ for 1 000 h; the inset is the SEM image after the stability test. f–i) Actual operation diagram of water splitting powered by wind, lithium cell, thermal, and sunlight.

Due to its dual activity in both OER and HER, FeCoNiWCuOOH@Cu electrode sheets were directly utilized as both anode and cathode catalysts, and an overall water‐splitting device was assembled in a 1.0 m KOH electrolyte to explore practical applications.^[^ ^53^ ^]^ As depicted in **Figure** **5**a,b, the FeCoNiWCuOOH@Cu||FeCoNiWCuOOH@Cu system requires only a battery voltage of 1.40 V to achieve a current density of 10 mA cm^−2^, significantly lower than the overpotential needed for the Pt/C/Cu||RuO_2_/Cu system (1.52 V @ 10 mA cm^−2^), consistent with the reported results.^[^ ^54^ ^]^ This performance surpasses the recently reported noble or non‐noble metal‐based catalysts (Figure 5d; Table S5, Supporting Information). Furthermore, H_2_ and O_2_ generated during the reaction process in the electrolytic cell were collected using a drainage method.^[^ ^55^ ^]^ The molar–time curves (Figure 5c) indicate that the actual volume ratio of collected H_2_ and O_2_ closely approximates the theoretical volume ratio of 2:1 in the electrocatalytic water‐splitting reaction. This suggests that the Faraday efficiency of the overall water‐splitting reaction is close to 100%. These findings undoubtedly demonstrate FeCoNiWCuOOH@Cu as a promising alkaline water‐splitting catalyst. Additionally, the FeCoNiWCuOOH@Cu||FeCoNiWCuOOH@Cu system exhibits remarkable durability. Following continuous electrolysis for 1000 h at 300 mA cm^−2^, the FeCoNiWCuOOH@Cu||FeCoNiWCuOOH@Cu system can still sustain 96% of the initial current density, with negligible performance degradation. Additionally, the FeCoNiWCuOOH@Cu||FeCoNiWCuOOH@Cu system demonstrates stable water splitting performance for over 500 h at a high current density of 500 mA cm^−2^, showcasing its strong competitiveness (Figure S35, Supporting Information). In comparison with the most advanced noble and non‐noble metal‐based catalysts previously reported (Table S5, Supporting Information), the prepared nanoporous double‐continuous pore structure Cu catalyst continues to demonstrate excellent water splitting stability, making it one of the best electrocatalysts for achieving overall water splitting. Evaluating the activity and stability of catalysts under practical application conditions is of great significance. Therefore, we reassessed the water electrolysis activity of the catalyst in a real water electrolyzer equipped with an anion exchange membrane. The results (Figure S36, Supporting Information) show that the water electrolyzer assembled with FeCoNiWCuOOH@Cu maintained good activity (1.532 V@10 mA cm^−2^) under the conditions of 30 wt.% KOH electrolyte and 60 °C and could run stably for at least 100 h at 500 mA cm^−2^. These results indicate that our catalyst exhibits acceptable performance and durability under practical application conditions.

After 1000 h of water splitting reaction, XRD, Raman, and SEM characterization were conducted to deepen the understanding of the stability of the catalytic system and the electrocatalytic reaction mechanism. XRD analysis (Figure S37a, Supporting Information) indicates that the peaks corresponding to the Cu skeleton in the XRD components notably decreased following the reaction. In contrast, the peak intensities related to Cu_2_O and CuO significantly increased. This suggests that after 1000 h of stability testing, the oxidation degree of the Cu skeleton has substantially improved. The apparent enhancement of Cu_2_O and CuO peaks in the Raman spectrum (Figure S37b, Supporting Information) further supports this observation. Subsequently, structural changes in the FeCoNiWCuOOH@Cu catalyst were analyzed via SEM analysis. It was observed (Figure S38, Supporting Information) that the FeCoNiWCuOOH@Cu catalyst maintained its nanoporous double‐continuous pore structure while developing a nanosheet‐like secondary structure on its surface. This phenomenon arises from the prolonged oxidation reaction of the FeCoNiWCuOOH@Cu electrode sheet, leading to further oxidation of the surface Cu skeleton. Additionally, the FeCoNiWCuO layer undergoes in situ oxidation to generate a highly active hydroxyl oxide protective layer, thereby preserving the nanoporous double‐continuous pore structure of the Cu skeleton.^[^ ^56^ ^]^ Subsequent element mapping and EDS spectra confirmed the relative stability of the overall element distribution and content in the FeCoNiWCuOOH@Cu electrode. These findings confirm the potential of the FeCoNiWCuOOH@Cu system as a comprehensive electrocatalyst for overall water splitting.

Establishing an effective external water‐splitting system is paramount in collecting, storing, and converting energy in the natural environment. Such a system not only helps reduce energy consumption and break free from dependence on electricity supply but also facilitates low‐cost, pollution‐free, and sustainable hydrogen production, which is significant.^[^ ^57^, ^58^ ^]^ Thus, a green energy hydrogen production system was introduced, wherein FeCoNiWCuOOH@Cu electrode sheets serve as components of OER/HER catalysts. As illustrated in Figure 5f–i, wind energy, lithium batteries, thermal energy, and solar energy were harnessed as driving forces for water electrolysis to generate hydrogen. Furthermore, the dynamic processes of efficient water splitting and gas accumulation, as demonstrated in Videos S1–S4 (Supporting Information), clearly demonstrate the excellent hydrogen production outcomes achieved by these green energy hydrogen production systems. These results indicate the broad application prospects of FeCoNiWCuOOH@Cu water‐splitting electrocatalysts in hydrogen production.

### 2.6 Catalytic Activity Mechanism

**Figure 6:**
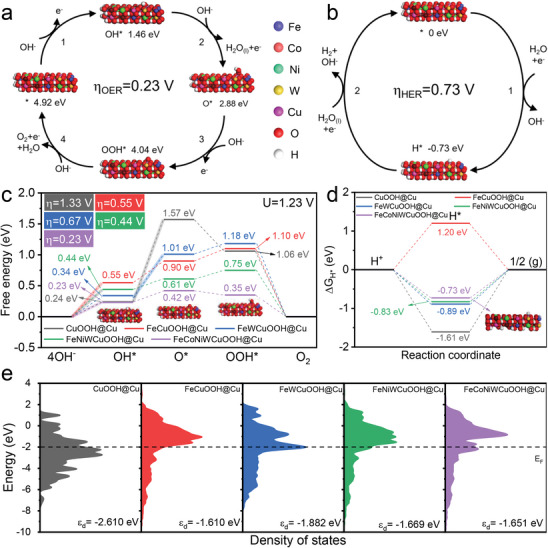
a,b) Schematic diagrams depicting the proposed mechanisms for (a) OER and (b) HER of FeCoNiWCuOOH@Cu. c,d) Free energy profiles for (c) OER with the set potential of 1.23 V and (d) HER. e) PDOS results for various samples.

In order to further elucidate the OER and HER mechanisms in alkaline FeCoNiWCuOOH@Cu solution, DFT calculations were conducted. For better representativeness, we used a CuOOH structure model with a size of 1.5 × 5 × 2 as the basis and additionally constructed four theoretical models with FeCuOOH, FeWCuOOH, FeNiWCuOOH, and FeCoNiWCuOOH (Figure S39, Supporting Information) to investigate the Gibbs free energy (Δ*G*) and local density of states (PDOS) of intermediates in OER and HER electroatalytic reactions. The first step for the OER and HER processes is to consider various sites to infer the most probable H_2_O adsorption sites on the samples. The results (Table S6, supporting information) indicate that Cu sites on CuOOH and FeCuOOH surfaces, as well as W sites on FeWCuOOH, FeNiWCuOOH, and FeCoNiWCuOOH surfaces, have the strongest adsorption energy for H_2_O. Therefore, the free energy evolution of the corresponding metal sites on the electrocatalyst surface for OER and HER was explicitly studied. As shown in **Figures** **6**a–d and S40 (Supporting Information), schematic diagrams of the OER and HER mechanisms and corresponding free energy in the proposed alkaline electrolyte are presented. The adsorption energy barriers of four essential intermediates (OH^*^, O^*^, OOH^*^, and H^*^) were analyzed through DFT calculations. For the OER process, the results in Figure 6c indicate that under the imposed potential of 1.23 V, the rate‐determining step (RDS) in the four‐electron process of CuOOH@Cu and FeWCuOOH@Cu is the formation of O^*^. At the same time, the RDS for FeCuOOH@Cu, FeNiWCuOOH@Cu, and FeCoNiWCuOOH@Cu is the adsorption of OH^−^. In addition, compared with CuOOH@Cu (1.33 V), FeCuOOH@Cu (0.55 V), FeWCuOOH@Cu (0.67 V), and FeNiWCuOOH@Cu (0.47 V), FeCoNiWCuOOH@Cu displays the lowest theoretical overpotential (0.23 V). For the HER process, compared with CuOOH@Cu (−1.61 V), FeCuOOH@Cu (1.20 V), FeWCuOOH@Cu (−0.89 V), and FeNiWCuOOH@Cu (−0.83 V), the hydrogen adsorption Gibbs free energy (Δ*G*_H*_) of FeCoNiWCuOOH@Cu is closer to the thermal neutral value (−0.73 V).^[^ ^36^, ^59^, ^60^ ^]^ These results indicate that the formation of high‐entropy structures is conducive to lowering their Gibbs free energy, thereby exhibiting significant dual functionality in promoting alkaline overall water splitting, consistent with the experimental results showing the optimal activity of FeCoNiWCuOOH@Cu (Figures 3a and 4a).

In order to determine the actual active sites of HER and OER, we conducted an in‐depth analysis of the results. A detailed analysis of the free energy diagram for OER (Figure 6c) reveals that in the 4OH^−^ → OH^*^ process, CuOOH@Cu and FeCoNiWCuOOH@Cu require relatively low energy absorption (0.24 and 0.23 eV). In comparison, FeCuOOH@Cu requires the highest energy absorption (0.55 eV). FeWCuOOH@Cu and FeNiWCuOOH@Cu require moderate energy absorption (0.34 and 0.44 eV), indicating that Co and Cu ions favor OH^−^ adsorption, while W and Ni show moderate performance, and Fe performs the worst. In the OH^*^ → O^*^ process, FeNiWCuOOH@Cu, and FeCoNiWCuOOH@Cu exhibit the lowest energy transition (0.17 and 0.22 eV), suggesting that Ni and Co facilitate OH^*^ dissociation. In the O^*^ → OOH^*^ process, FeCoNiWCuOOH@Cu has already achieved energy reduction (−0.07 eV), indicating that Co promotes the conversion of O^*^ to OOH^*^. Although CuOOH@Cu shows greater energy reduction (−0.51 eV) during this reaction, the high energy barrier in the OH^*^ → O^*^ process (1.33 eV) hinders the further OER process of Cu. Considering the rate‐determining steps and required energy for each catalyst, Co is the primary active element in the OER process. Cu contributes more to OH^−^ dissociation, while Ni facilitates OH^*^ dissociation. An in‐depth study of the free energy diagram for HER (Figure 6d) reveals that CuOOH@Cu and FeCuOOH@Cu exhibit the most extreme Δ*G*_H*_ values (−1.61 and 1.20 eV). In contrast, FeWCuOOH@Cu, FeNiWCuOOH@Cu, and FeCoNiWCuOOH@Cu have more moderate Δ*G*_H*_ values (−0.89, −0.83, and −0.73 eV). Additionally, we calculated the adsorption energies of H_2_O for each sample (Figure S41, Supporting Information). The results show that CuOOH@Cu has the best H_2_O adsorption performance (−4.89 eV), leading to more incredible difficulty in subsequent reactions. While FeCuOOH@Cu has the most suitable water adsorption energy (−1.52 eV), its high water dissociation barrier limits its activity. FeWCuOOH@Cu, FeNiWCuOOH@Cu, and FeCoNiWCuOOH@Cu have moderate water adsorption energies (−1.83, −1.52, and −1.74 eV), which are beneficial for the subsequent processes. These results indicate that W, Ni, and Co are conducive to the overall HER reaction, while Cu plays a significant role in H_2_O adsorption.

Further calculations were conducted to determine the density of states (DOS) for each sample (Figure 6h). The results revealed that CuOOH@Cu exhibited a lower d‐band center (ε_d_) value (−2.610 eV). Upon introduction of Fe atoms, the ε_d_ value increased to −1.610 eV (FeCuOOH@Cu sample). With the introduction of W atoms (FeWCuOOH@Cu sample), ε_d_ fell to −1.882 eV. The subsequent introduction of Ni and Co atoms resulted in varying degrees of increase in ε_d_ (FeNiWCuOOH@Cu sample: −1.669 eV, FeCoNiWCuOOH@Cu sample: −1.651 eV). A deeper analysis of this trend of change was influenced by atomic radii. Due to the introduction of various metal elements (Fe, Co, Ni, and W), different degrees of increased/decreased bond length and strain occurred within the CuOOH structure (Figure S42, Supporting Information), leading to the narrowing/widening of ε_d_. Consequently, the occupation of antibonding orbitals weakened/enhanced, affecting the adsorption strength of reaction intermediates.^[^ ^61^ ^]^ According to the ε_d_ theory,^[^ ^62^ ^]^ ε_d_ values indicating moderate adsorption strength suggest more favorable adsorption/desorption, thus facilitating rapid overall water splitting. The moderate ε_d_ value of FeCoNiWCuOOH@Cu is beneficial for enhancing the catalytic performance of both OER and HER. This also suggests that HEO catalysts with optimal adsorption energy can be designed by introducing different metal elements.

## 3 Conclusions

In summary, a novel strategy combining liquid metal dealloying and electrodeposition has been developed to design FeCoNiWCuOOH@Cu heterostructure HEOs nanocomposite dual‐functional electrocatalysts with a double‐continuous pore structure. The optimized FeCoNiWCuOOH@Cu electrode demonstrates excellent activity in both OER and HER, achieving a current density of 10 mA cm^−2^ with ultra‐low overpotentials of only 200 and 18 mV, respectively. The corresponding Tafel slopes are as low as 24 and 14 mV dec^−1^, respectively. Moreover, the overall water splitting device assembled with FeCoNiWCuOOH@Cu electrode sheets operates at a low battery voltage of 1.40 V, achieving a current density of 10 mA cm^−2^, and exhibits high stability for over 1000 h of operation at 300 mA cm^−2^. Notably, the formation of the double‐continuous pore structure enhances the exposure of active sites, while high‐entropy engineering enhances the intrinsic activity of the catalysts. The synergistic effect of multiple elements and the precise regulation of nanostructures provide new impetus for improving the performance of high‐entropy catalysts. Furthermore, the HEO layer formed on the surface of the Cu skeleton effectively isolates atomic diffusion, endowing the FeCoNiWCuOOH@Cu electrode sheets with long‐term durability. DFT calculations also elucidate the strong coupling effect of multi‐element interactions on OER/HER activity. The successful demonstration of green energy hydrogen production systems opens up new possibilities for the practical large‐scale application of electrocatalytic water splitting technology. This work is expected to advance the development of externally driven large‐current water‐splitting systems.

## 4 Experimental Section

### Materials

Cu (99.9%) and Al (99.9%) were obtained from Beijing Zhongke Yannuo New Materials Technology Co., Ltd. Hydrochloric acid (HCl, 37 wt.%) was purchased from Honeywell International Inc. Iron chloride (FeCl_3_, A.R.) was provided by Advanced Technology & Industrial Co., Ltd. Cobaltous nitrate hexahydrate (Co(NO_3_)_2_·6H_2_O, A.R.), anhydrous citric acid (C_6_H_8_O_7_, A.R.), Pt/C (A.R.), potassium hydroxide (KOH, A.R.) and potassium phosphate dibasic anhydrous (K_2_HPO_4_, A.R.) were purchased from Shanghai Meryer Chemical Technology Co., Ltd. Nickel chloride hexahydrate (NiCl_2_·6H_2_O, A.R.) was obtained from Beijing InnoChem Science & Technology Co., Ltd. Sodium tungstate dihydrate (Na_2_WO_4_·2H_2_O, A.R.) and ruthenium oxide (RuO_2_, A.R.) were provided by Shanghai Aladdin Biochemical Technology Co., Ltd. Nafion solution (5%) and sodium phosphate monobasic dihydrate (NaH_2_PO_4_·2H_2_O, A.R.) were purchased from Sigma–Aldrich, USA. Ethanol (C_2_H_6_O, A.R.) was provided by Anaqua Global International Inc. Limited. Deionized (DI) water was obtained from an OmniaLab ED+ 40 Ultrapure water system.

### Synthesis of FeCoNiWCuOOH@Cu System Electrocatalysts

A precursor of Cu_20_Al_80_ alloy sheet with a 1 cm × 1 cm × 250 µm thickness was prepared by arc melting Cu and Al metals in N_2_ atmosphere, followed by cooling and cutting procedures. Then, it was immersed in a 2 m HCl solution for 4 h for chemical dealloying to remove Al. The dealloyed sample was rinsed with ultrapure water to remove residual chemicals in the nanopores and obtain a nanoporous Cu framework. FeCl_3_ (1 mmol), Co(NO_3_)_2_·6H_2_O (1 mmol), NiCl_2_·6H_2_O (1 mmol), and 5 mmol anhydrous citric acid were dissolved in 30 mL of solution (15 mL of H_2_O and C_2_H_6_O each), denoted as solution A. Na_2_WO_4_·2H_2_O (3.5 mmol) was dissolved in 20 mL of H_2_O, denoted as solution B. Solution B was added to solution A to obtain the electroplating solution. A three‐electrode system was assembled using the cleaned sample, the graphite rod electrode, and the saturated calomel electrodes as working, counter, and reference electrodes, respectively. At a potential of −4 V (relative to the saturated calomel electrode), FeCoNiWCuO NS were electrodeposited on a nanoporous Cu framework. Subsequently, a three‐electrode system was assembled using a Hg/HgO electrode as the reference electrode and 1 m KOH solution as the electrolyte. FeCoNiWCuO NS were subjected to 10 times CV tests at a scanning rate of 20 mV s^−1^ in the potential range of 0–1.5 V, and the FeCoNiWCuOOH@Cu catalyst was ultimately obtained. Only the relevant composition of the electroplating solution is modified in the preparation process of FeCuOOH@Cu, FeWCuOOH@Cu, and FeNiWCuOOH@Cu catalysts, and the other preparation processes are the same as those of the FeCoNiWCuOOH@Cu catalyst. The electroplating process is eliminated in the preparation process of the CuOOH@Cu catalyst, and other processes are the same as those of the FeCoNiWCuOOH@Cu catalyst. By mixing commercially available Pt/C and RuO_2_ nanocatalysts and Nafion in a solution containing ethanol (20%) and water (80%) and dropwise coating them onto the nanoporous Cu framework, Pt/C and RuO_2_ electrodes loaded on the nanoporous Cu framework were prepared, respectively.

### Characterization

TEM, HRTEM, SAED, and EDX analyses were conducted with JEOL JEM‐2100F equipment. The surface morphologies were observed by SEM (S‐4800) equipped with an EDS detector at 5 and 30 kV acceleration voltages. The crystal structures of the alloy sheets were analyzed by a D2 PHASER XE‐T X‐ray Diffractometer System equipped with Cu Kα radiation (*λ* = 1.5406 Å). Raman spectroscopy was employed in a WITec alpha 300 R Raman System with a 532 nm laser excitation source. The XPS data were collected by PHI 5000 Versaprobe system with a monochromatic Al Kα radiation source. All binding energies were calibrated by shifting the C 1s peak to 284.8 eV. The contact angles of electrode tabs were tested by DataPhysics Contact Angle Tester. The specific surface area of the catalysts was obtained through BET testing using the 3FLEX 3500 Multi‐Port High‐Throughput Gas Adsorption Analyzer.

### Electrochemical Measurements

All electrochemical tests were conducted in a standard three‐electrode system connected to the Chenhua CHI760E electrochemical workstation. The reference electrode was calibrated before the performance testing. All potentials have undergone 80% iR correction. Before LSV testing, 20 CV cycles were performed at a scanning rate of 5 mV s^−1^ to remove the passivation surface of the working electrode. A Hg/HgO electrode and graphite rod electrode are used as the reference and counter electrodes in 1 m KOH solution. For HER, OER, and water splitting experiments, LSV scans were performed at a scan rate of 5 mV s^−1^ and potential intervals from −1.6 to −0.9 V, 0 to 1.5 V, and 0 to 1.5 V, respectively. The potentials in 1 M KOH solution were converted to the corresponding potentials versus reversible hydrogen electrode (vs RHE) using Equation (1):

(1) $$E_{\mathrm{RHE}}=\;E_{\mathrm{Hg}/\mathrm{HgO}}+0.098+0.059\;\times \;\mathrm{pH}$$

where *E*_RHE_ and *E*_Hg/HgO_ are the potentials versus RHE and measured potentials versus Hg/HgO reference electrode, respectively.

The ECSAs of the catalysts were calculated from the C_dl_ profiles using Equation (2):^[^ ^63^, ^64^ ^]^

(2) $$\mathrm{ECSA}\;=\;\left(C_{dl\;}/\;C_{s}\right)A_{geo}$$

where C_s_ = 0.040 mF cm^−2^ and *A*_geo_ (0.5 cm^2^) is the actual geometric area of the alloy sheets exposed to the electrolyte.

First, The currents without the Faradaic process were measured. Then, *C*_dl_ values were calculated from the half slope of the linearly fitted curves of the capacitive current and plotted as a function of the scan rate.^[^ ^65^ ^]^

To calculate TOF values of the catalysts, CV scans were performed in 1 m phosphate buffer saline (PBS) solution with a scanning voltage of 50 mV s^−1^ from −0.2 to 0.6 V (vs RHE). The TOF values were calculated based on Equations (3) and (4):^[^ ^66^ ^]^

(3) TOF=I/2nF

(4) n=Q/2F

Among them, *I* refers to the current (in A) during the LSV measurement process, *n* represents the number of active sites (in mol), *F* is the Faraday constant (in 96 500 C mol^−1^), and *Q* is the number of voltammetric charges (in C).

The EIS measurements were conducted at open circuit potential in the frequency range from 100 kHz to 0.01 Hz.

### Computational Methods

The electronic optimizations were achieved by employing spin‐polarized DFT calculations using a facet‐wave method implemented in the Vienna Ab Initio Simulation Package (VASP) code.^[^ ^67^ ^]^ The core‐valence interaction was described using the projection augmented wave (PAW) method. At the same time, the electron exchange and correlation energy were accounted for using generalized gradient approximation (GGA) with the Perdew–Burke–Ernzerhof (PBE) function.^[^ ^68^ ^]^ A cutoff energy of 450 eV was chosen, and spin‐polarization calculations were performed for all structural optimizations. Using Löwdin‐orthogonalized atomic projectors, calculations were carried out at the GGA+U level with the Hubbard U correction. The U values applied were 3 eV for Co, 4 eV for Cu, and 8 eV for both Ni and Fe.^[^ ^20^ ^]^

The OER/HER free energy diagrams of the FeCoNiWCuOOH system were calculated, and the total OER/HER equations were acquired by Equations (5)–(9):

For OER:

(5) $$4OH^{-}\;\to \;OH^{*}+\;3OH^{-}\;+\;e^{-}$$

(6) $$\to \;O^{*}+H_{2}O\;+\;2OH^{-}\;+\;2e^{-}$$

(7) $$\to \;\mathrm{HO}O^{*}+\;H_{2}O\;+\;OH^{-}\;+\;3e^{-}$$

(8) $$\to \;O_{2}\;+\;2H_{2}O\;+\;4e^{-}$$

For HER:

(9) $$H^{+}\;+\;e^{-}\to \;1/2H_{2}$$

The free energy (chemical potential) of each hydrogen atom in the initial and final states was considered equal in the equilibrium state. The free energies of OER/HER intermediates were computed based on Equation (10):

(10) ΔG=ΔE+ΔZPE−TΔS

where Δ*G*, Δ*S*, and ΔZPE represent the binding energy, entropy change, and zero‐point energy change at 298.15 K, respectively.^[^ ^69^ ^]^

## Conflict of Interest

The authors declare no conflict of interest.

## Supporting information

Supporting Information

Supplemental Video 1

Supplemental Video 2

Supplemental Video 3

Supplemental Video 4

## Data Availability

The data that support the findings of this study are available from the corresponding author upon reasonable request.
