# Supplementary material for: A High‐Entropy Oxyhydroxide with a Graded Metal Network Structure for Efficient and Robust Alkaline Overall Water Splitting
Source: Adv Sci (Weinh). 2024 Aug 13;11(39):2406008. doi: 10.1002/advs.202406008 (PMC11496996; doi:10.1002/advs.202406008)
Supplement: Supplementary file 1 — Supporting Information [file ADVS-11-2406008-s005.pdf]

ADVANCED SCIENCE
Open Access

Supporting Information

for *Adv. Sci.*, DOI 10.1002/advs.202406008

A High-Entropy Oxyhydroxide with a Graded Metal Network Structure for Efficient and Robust Alkaline Overall Water Splitting

Chen-Xu Zhang, Di Yin, Yu-Xuan Zhang, Yu-Xiang Sun, Xiao-Jin Zhao, Wu-Gang Liao and Johnny C. Ho**

Supporting Information

A High-Entropy Oxyhydroxide with a Graded Metal Network Structure for Efficient and Robust Alkaline Overall Water Splitting

Chen-Xu Zhang, Di Yin, Yu-Xuan Zhang, Yu-Xiang Sun, Xiao-Jin Zhao, Wu-Gang Liao, and Johnny C. Ho**

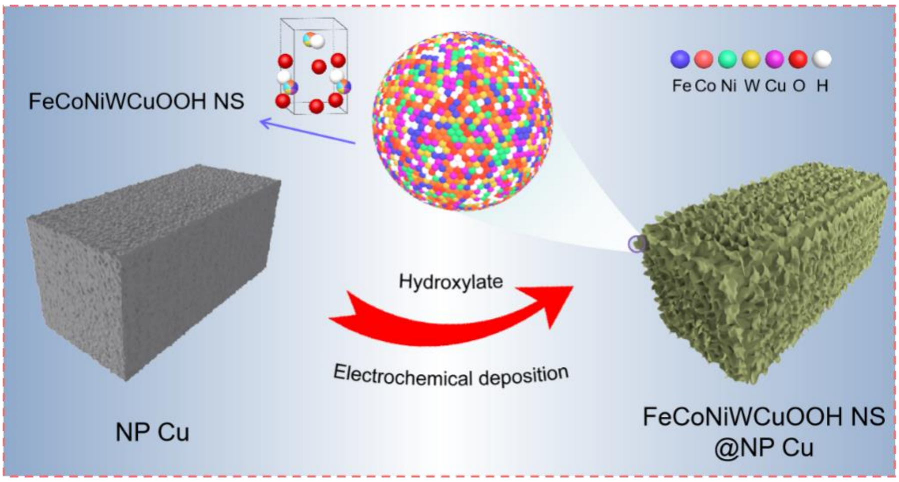

Figure S1. Synthesis diagram of FeCoNiWCuOOH Ns.

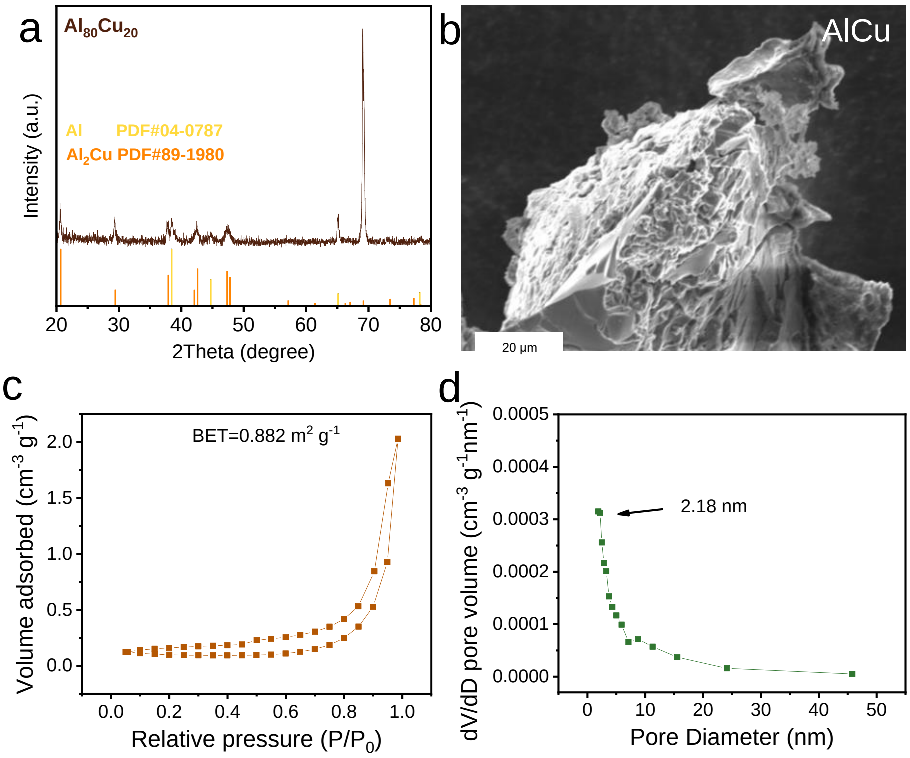

Figure S2. (a) XRD pattern, (b) SEM image, (c) N_2 sorption/desorption isotherm, and (d) corresponding pore size distribution curve of AlCu.

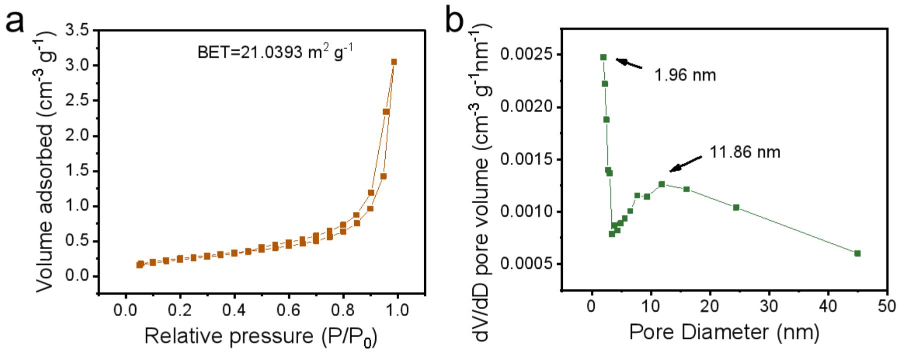

Figure S3. (a) N_2 sorption/desorption isotherm and (b) corresponding pore size distribution curve of FeCoNiWCuOOH@Cu.

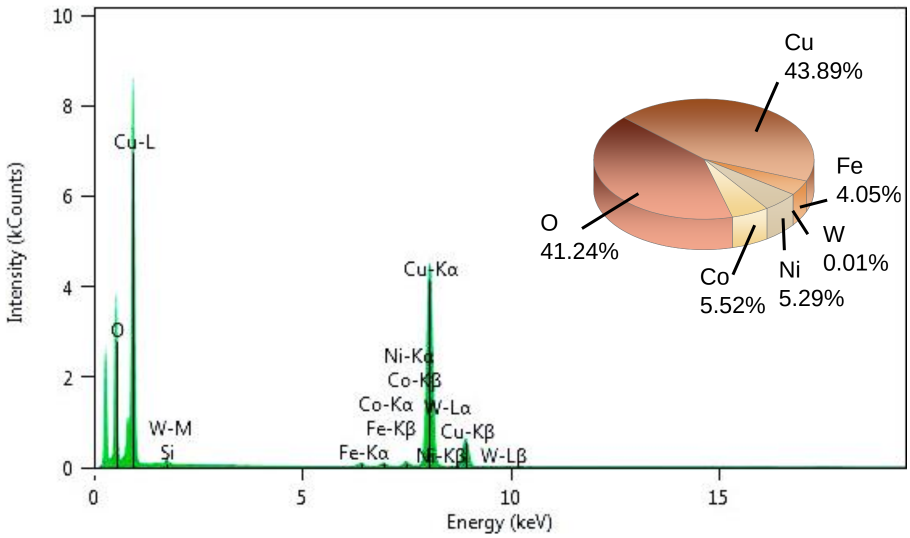

Figure S4. EDS spectra, the inset is the elemental map of FeCoNiWCuOOH@Cu.

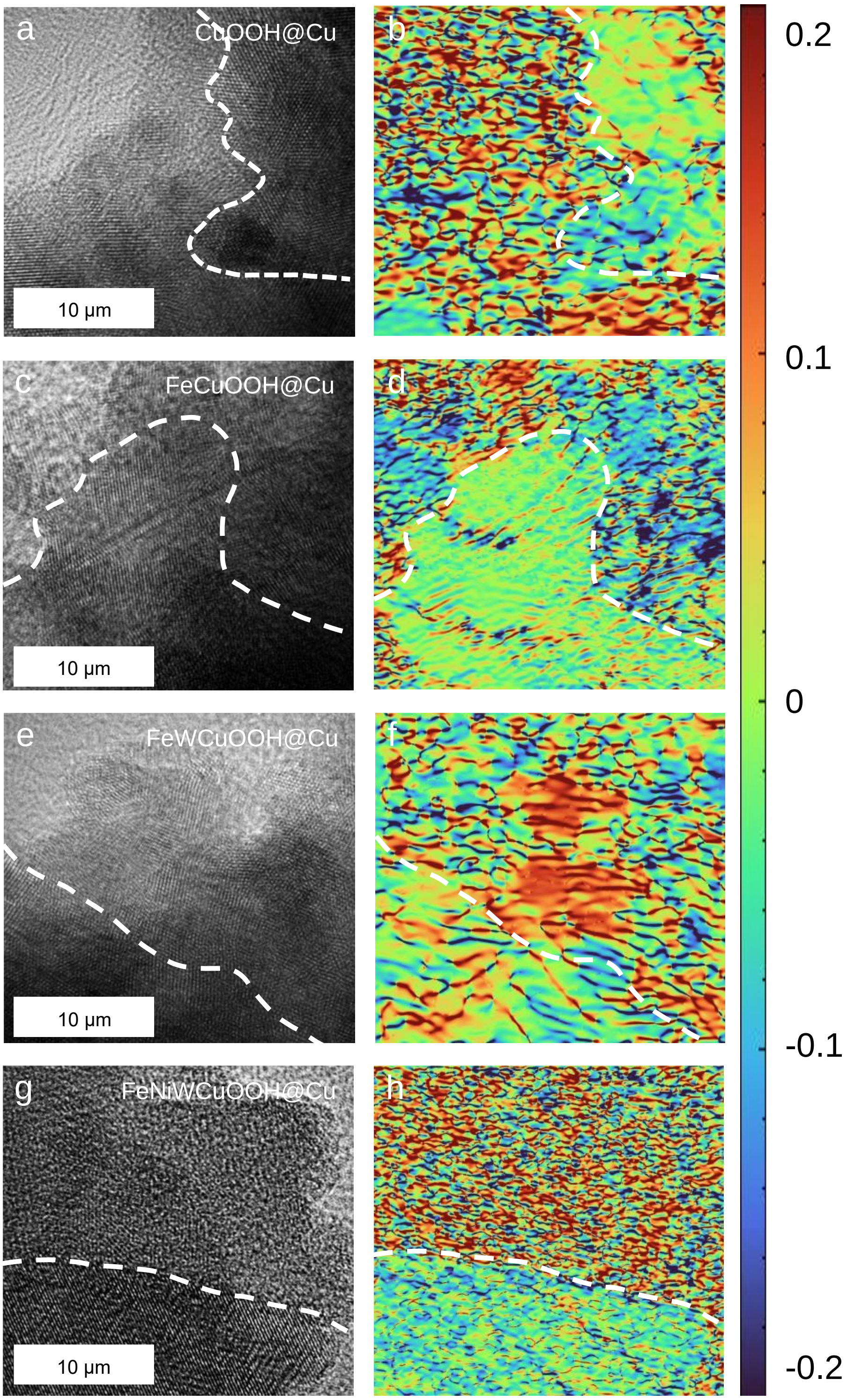

Figure S5. (a, c, e, g) HRTEM images, (b, d, f, h) strain distributions along E_{xy} of different samples. The compressive strain of (b, d, f, h) shifted from green to dark blue, and the tensile strain shifted from bright yellow to red.

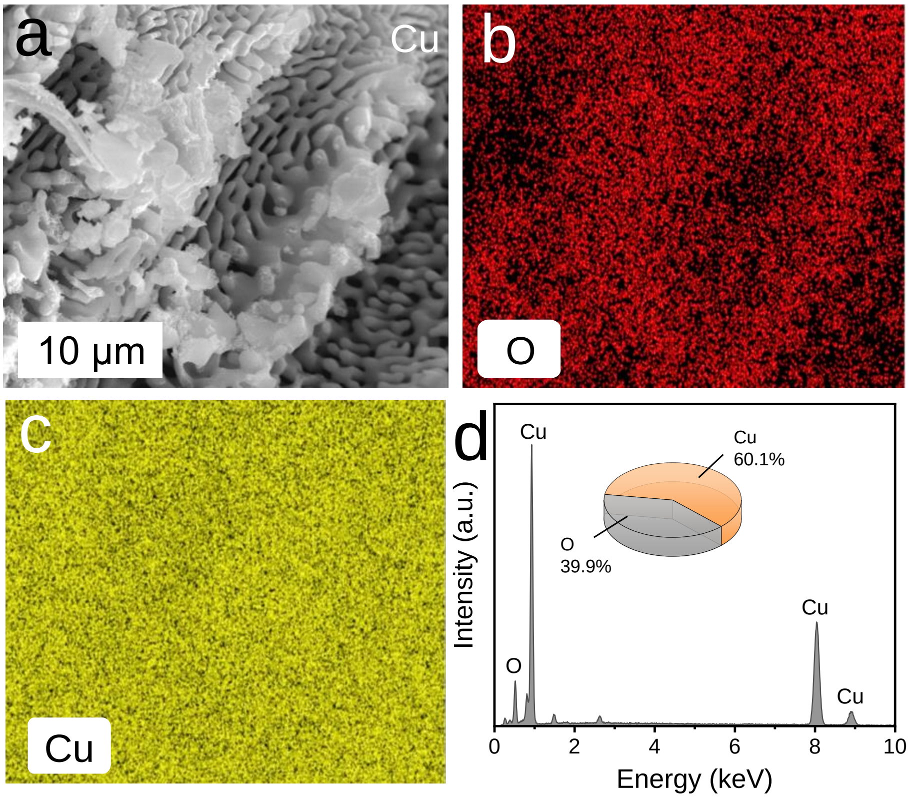

Figure S6. (a) SEM image, (b, c) The corresponding EDS elemental mappings, and (d) EDS spectra of Cu. The inset of (d) is the elemental map.

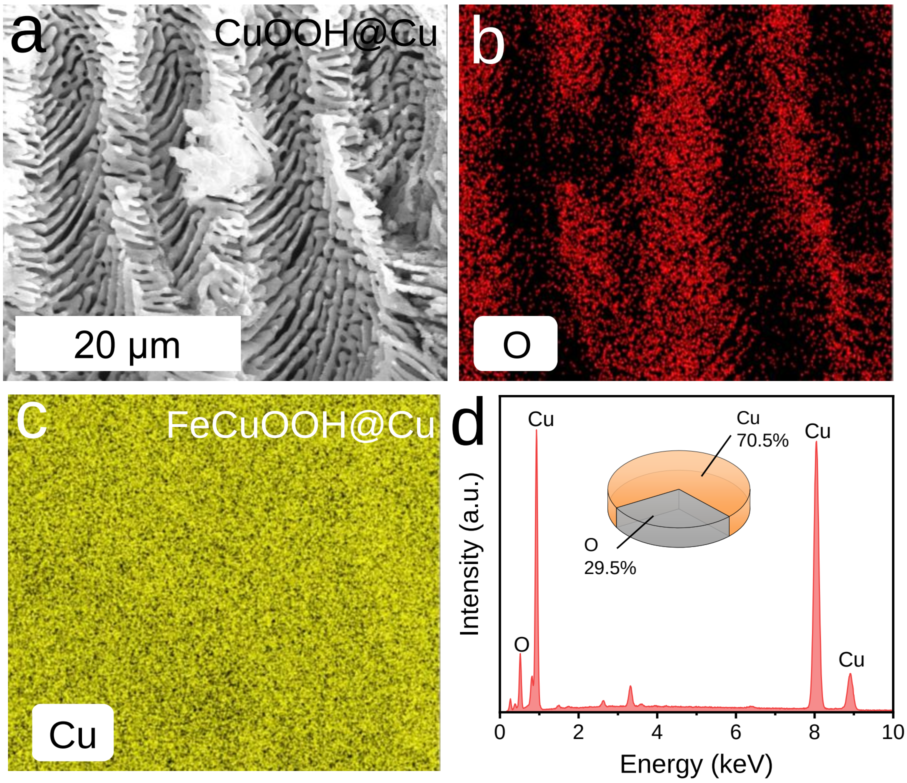

Figure S7. (a) SEM image, (b, c) The corresponding EDS elemental mappings, and (d) EDS spectra of CuOOH@Cu. The inset of (d) is the elemental map.

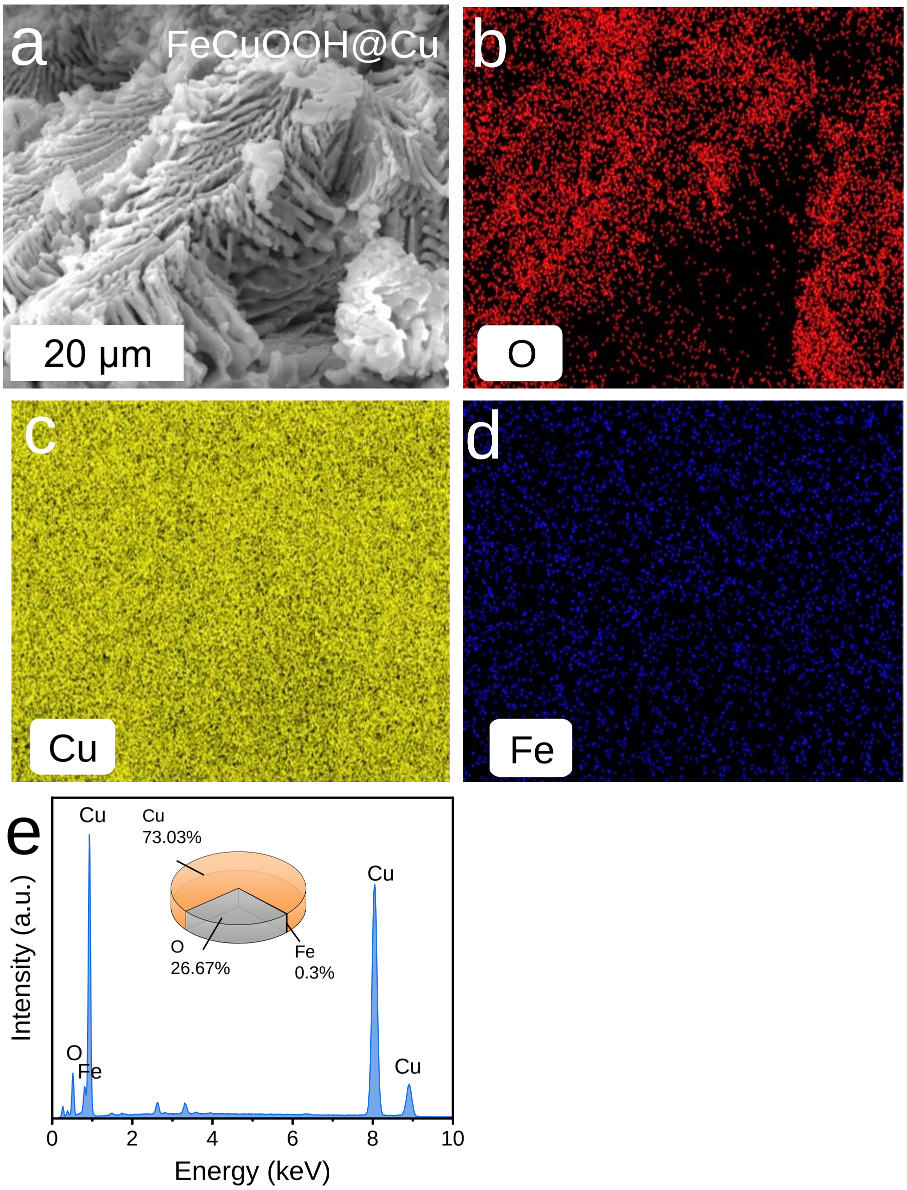

Figure S8. (a) SEM image, (b-d) The corresponding EDS elemental mappings, and (e) EDS spectra of FeCuOOH@Cu. The inset of (e) is the elemental map.

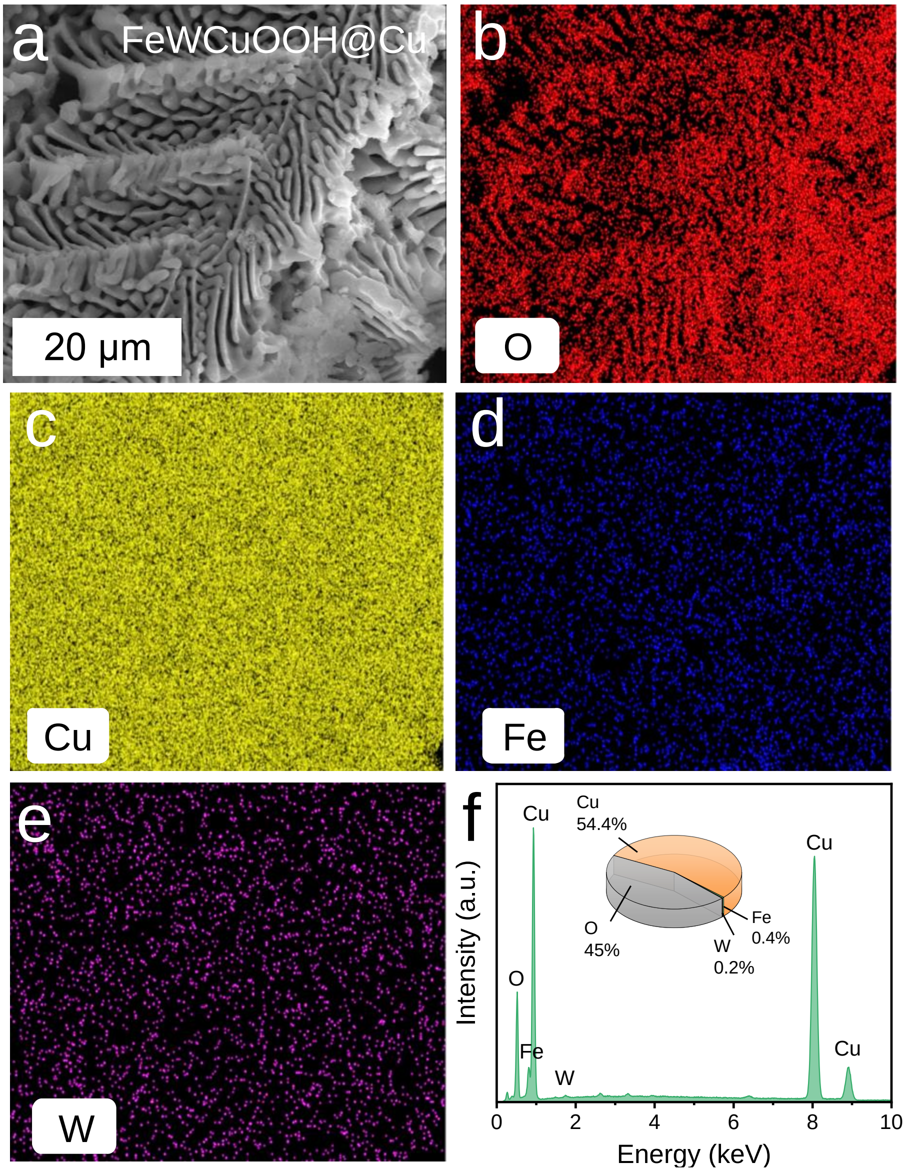

Figure S9. (a) SEM image, (b-e) The corresponding EDS elemental mappings, and (f) EDS spectra of FeWCuOOH@Cu. The inset of (f) is the elemental map.

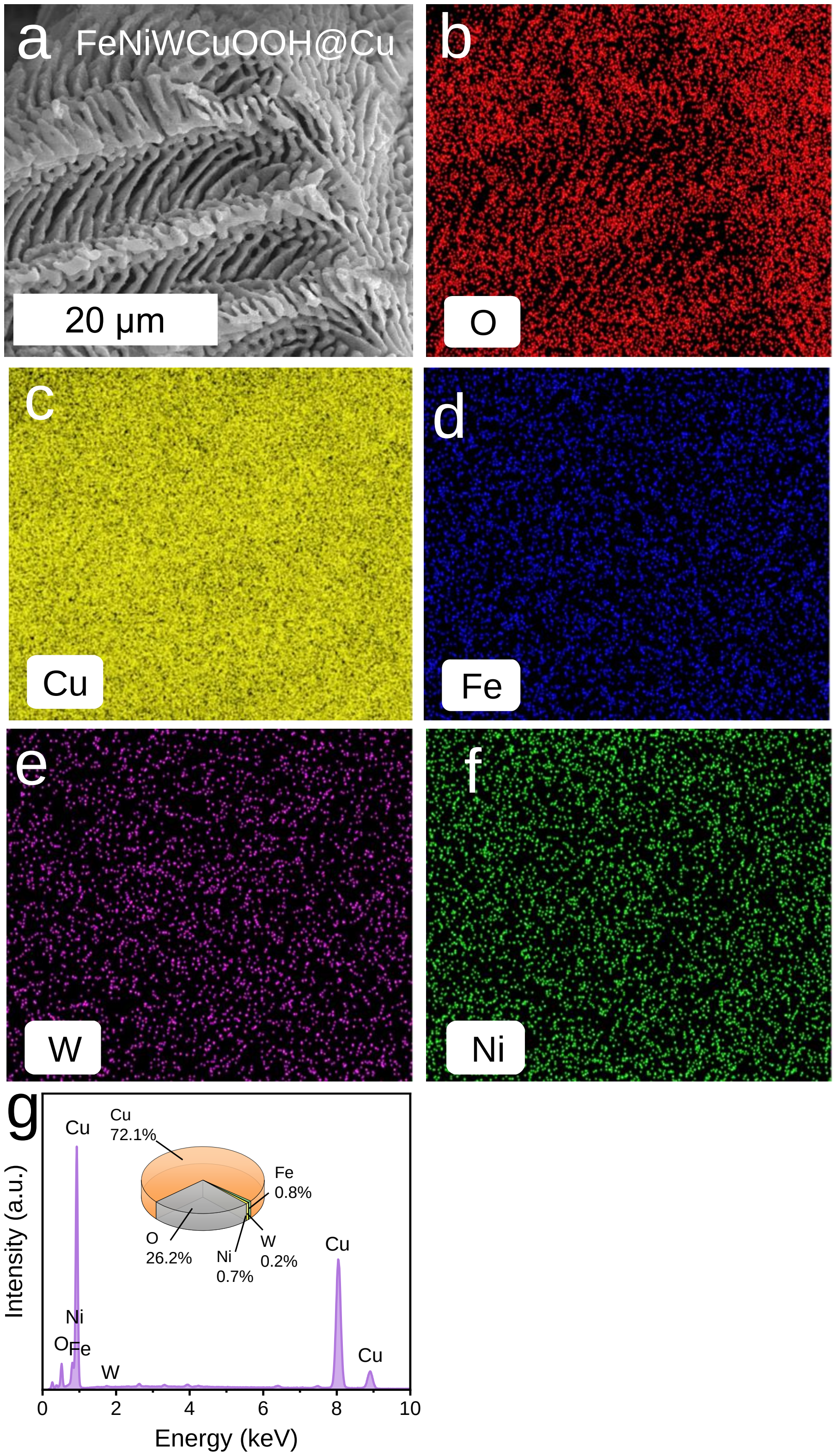

Figure S10. (a) SEM image, (b-f) The corresponding EDS elemental mappings, and (g) EDS spectra of FeNiWCuOOH@Cu. The inset of (g) is the elemental map.

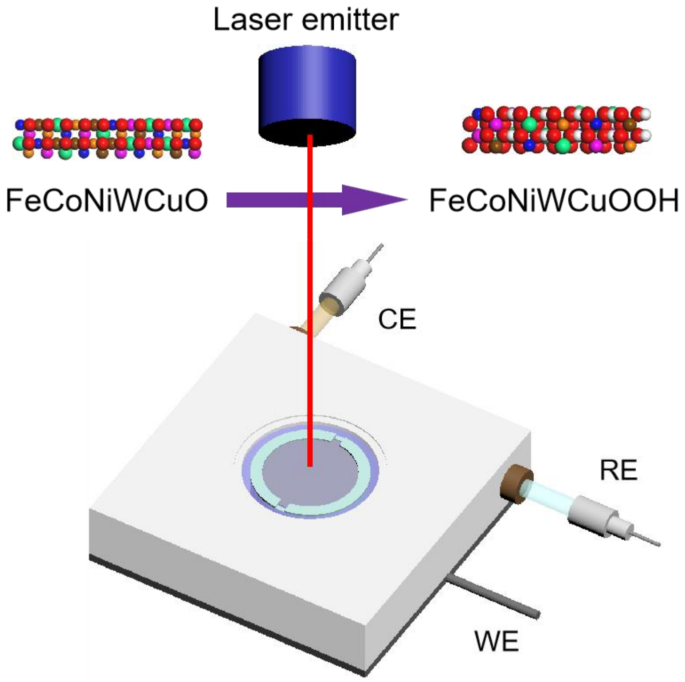

Figure S11. Schematic illustration of *in-situ* electrochemical Raman spectra of phase transformation of FeCoNiWCuOOH@Cu during the electrochemical synthesis process.

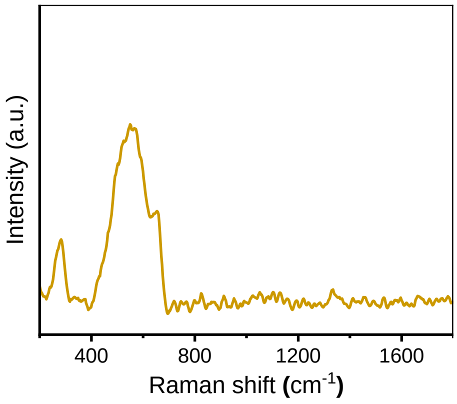

Figure S12. Raman pattern of FeCoNiWCuOOH@Cu.

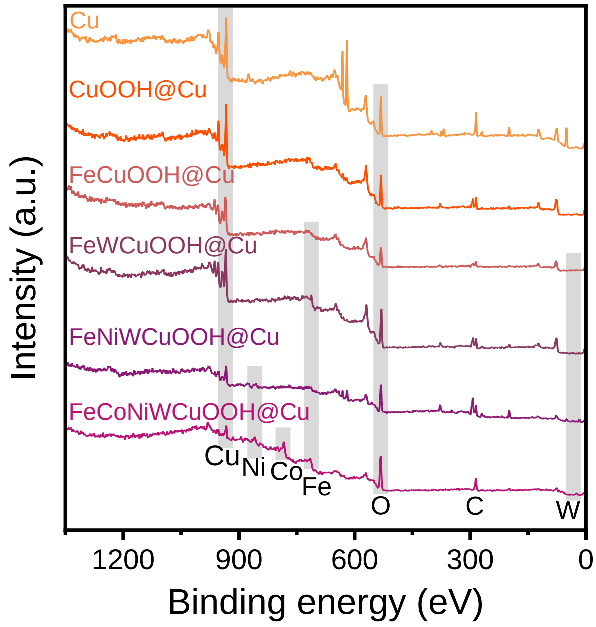

Figure S13. XPS survey spectra of Cu, CuOOH@Cu, FeCuOOH@Cu, FeWCuOOH@Cu, FeNiWCuOOH@Cu, and FeCoNiWCuOOH@Cu.

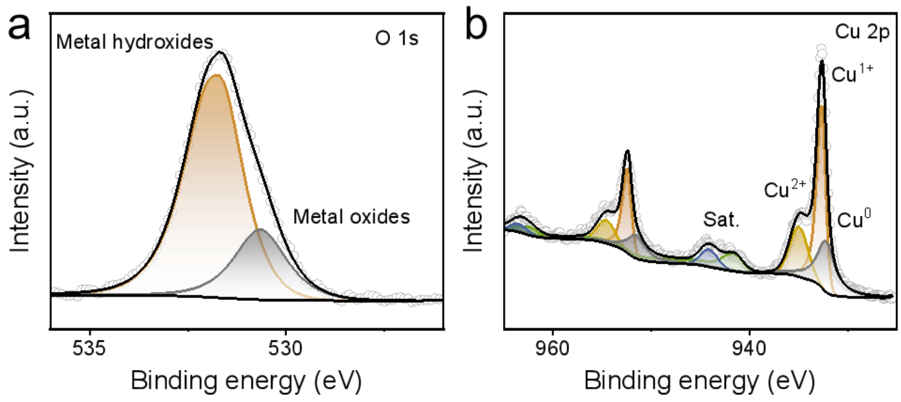

Figure S14. (a, b) High-resolution XPS results of Cu.

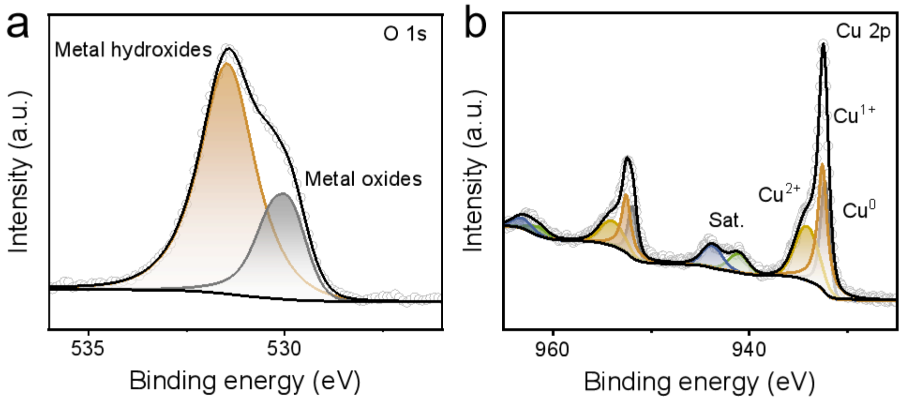

Figure S15. (c, d) High-resolution XPS results of CuOOH@Cu.

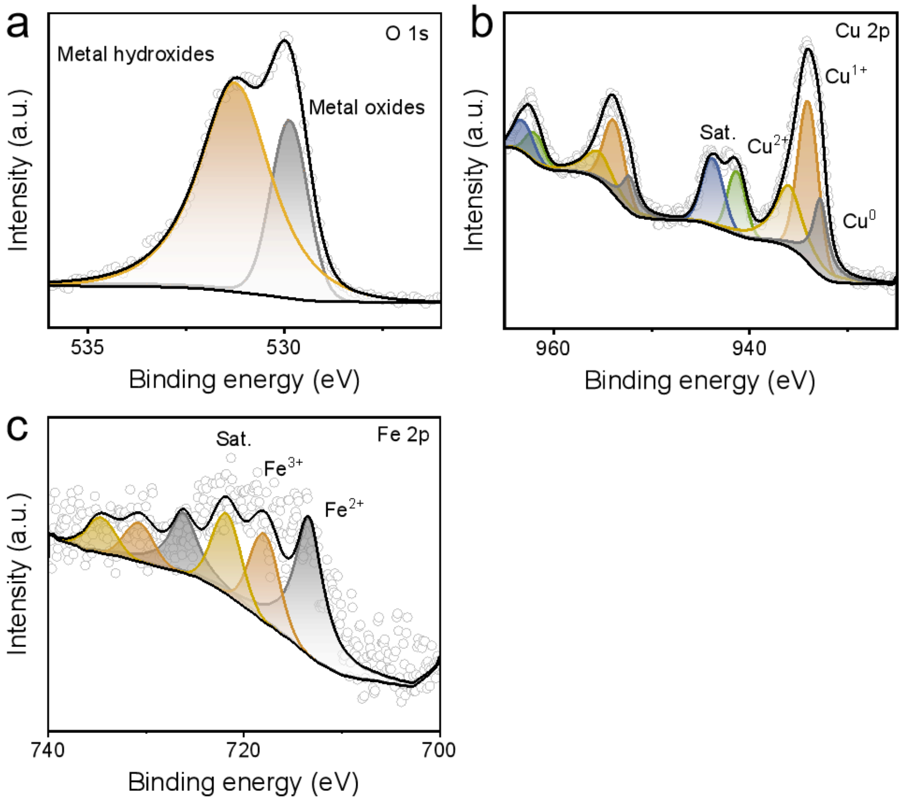

Figure S16. (a-c) High-resolution XPS results of FeCuOOH@Cu.

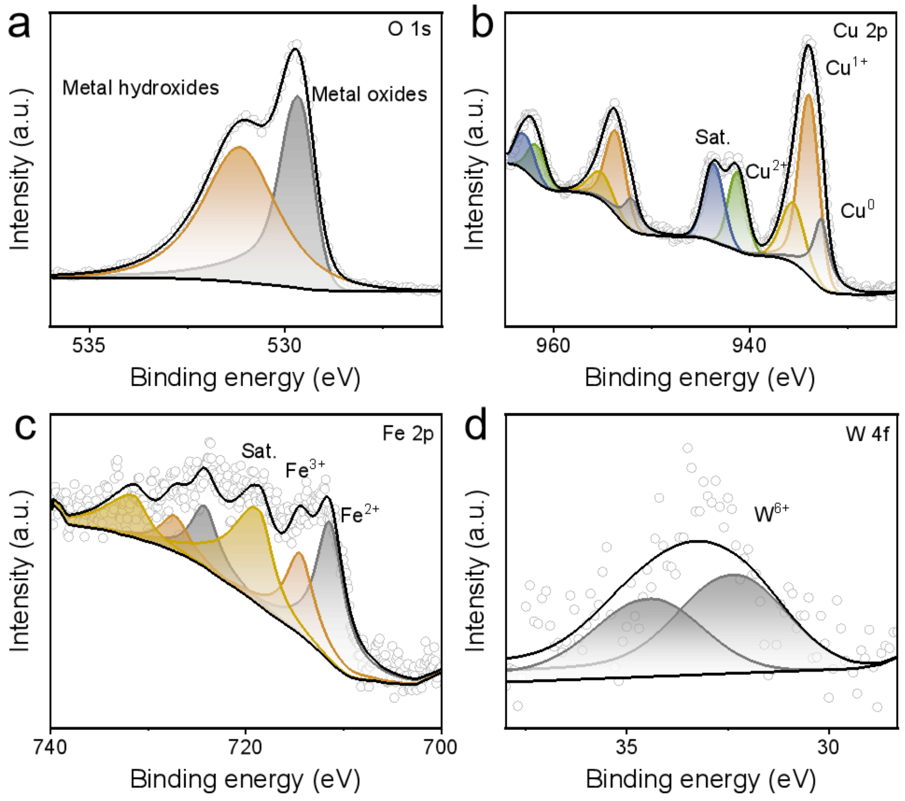

Figure S17. (a-d) High-resolution XPS results of FeWCuOOH@Cu.

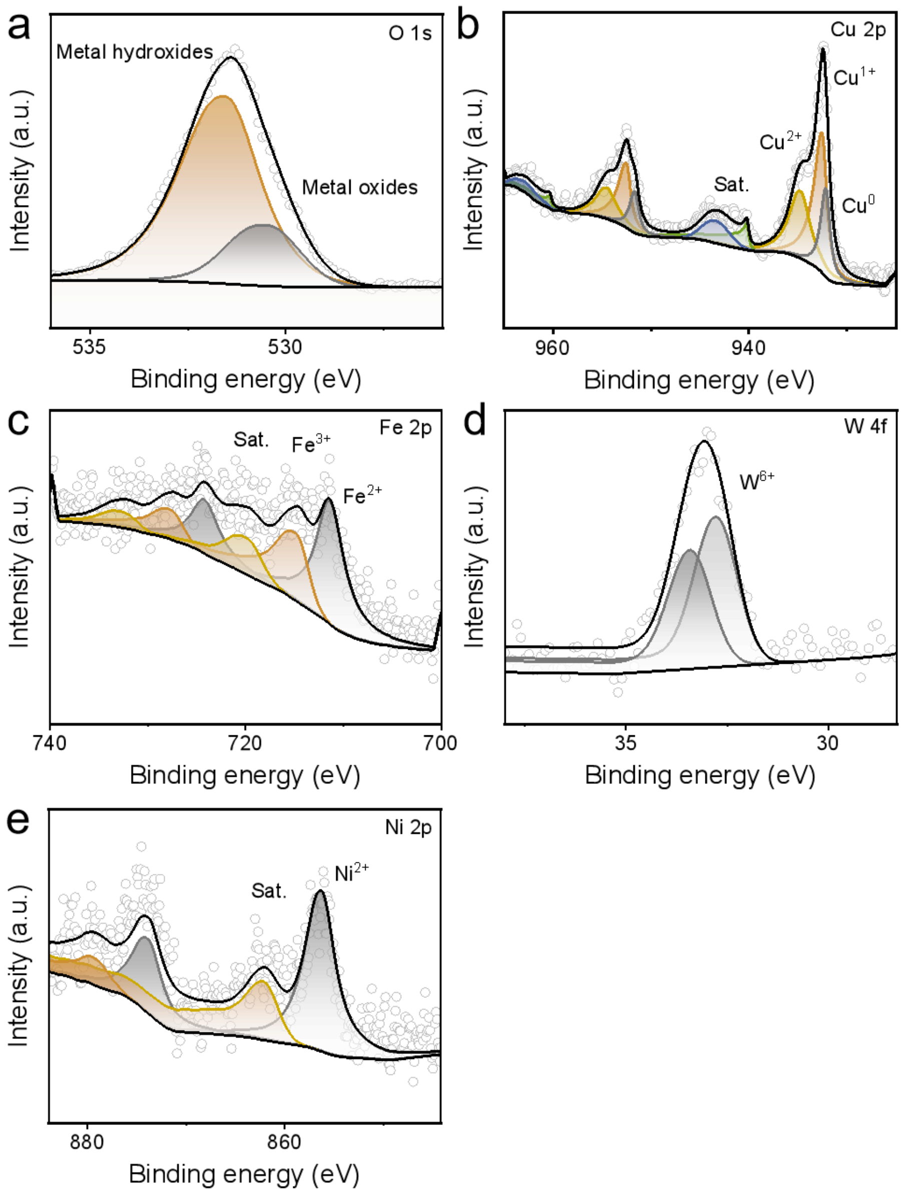

Figure S18. (a-e) High-resolution XPS results of FeNiWCuOOH@Cu.

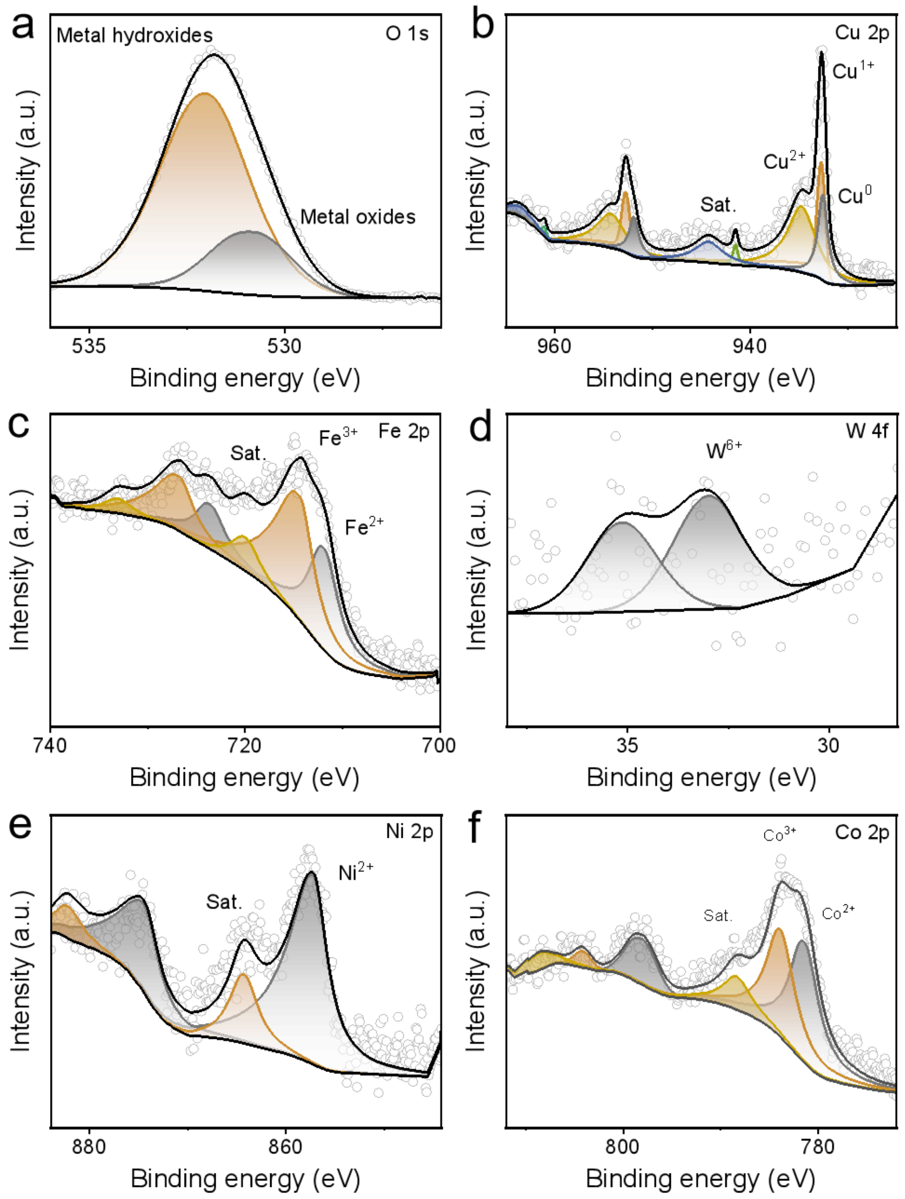

Figure S19. (a-f) High-resolution XPS results of FeCoNiWCuOOH@Cu.

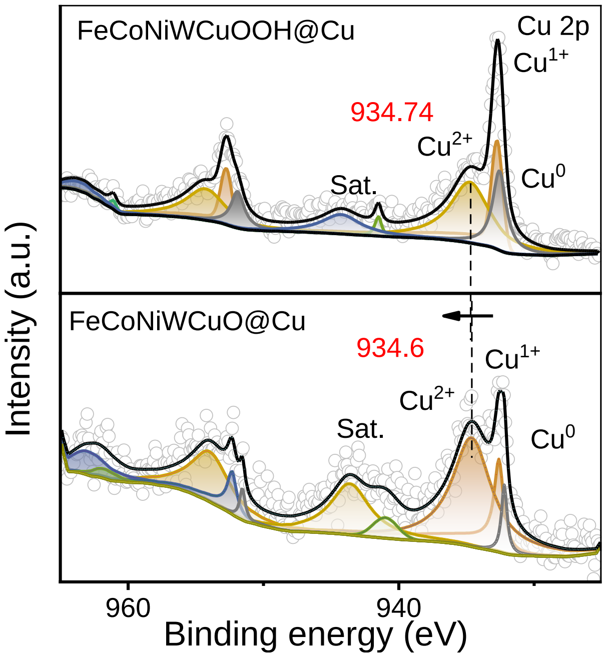

Figure S20. High-resolution XPS result of FeCoNiWCuO@Cu for Cu 2p.

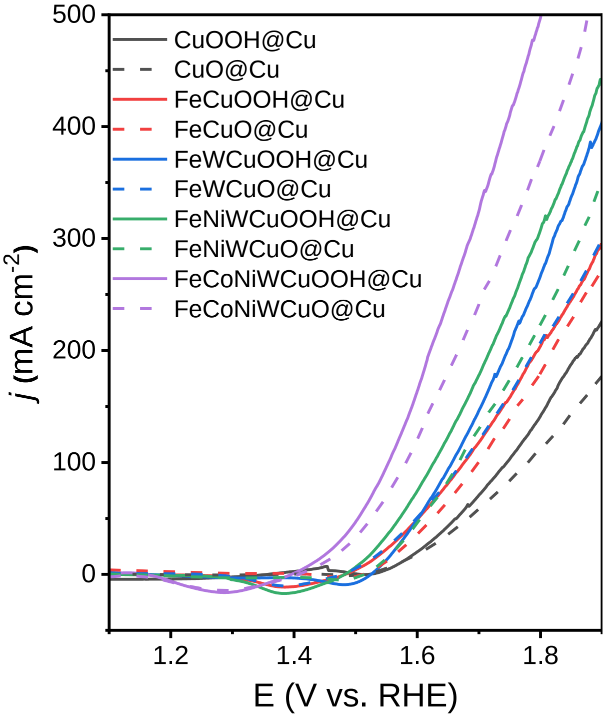

Figure S21. OER polarization curves of different samples before and after hydroxylation treatment.

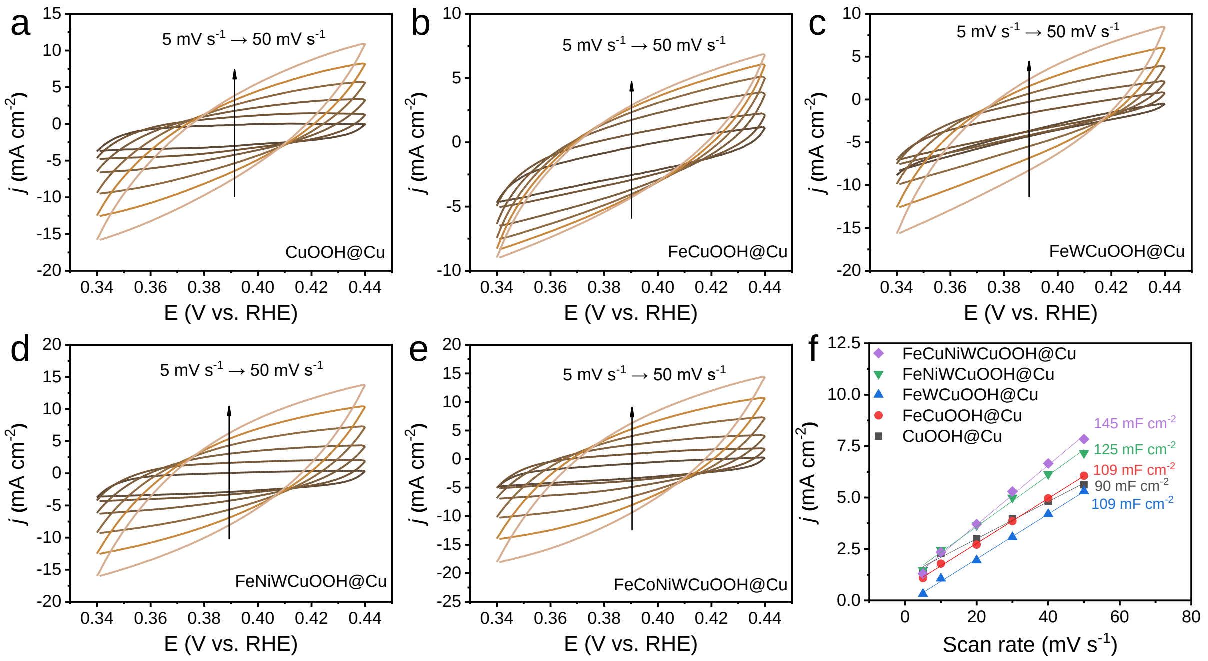

Figure S22. CV curves of (a) CuOOH@Cu, (b) FeCuOOH@Cu, (c) FeWCuOOH@Cu, (d) FeNiWCuOOH@Cu and (e) FeCoNiWCuOOH@Cu. (f) Capacitive current density versus scan rate for CuOOH@Cu, FeCuOOH@Cu, FeWCuOOH@Cu, FeNiWCuOOH@Cu, and FeCoNiWCuOOH@Cu.

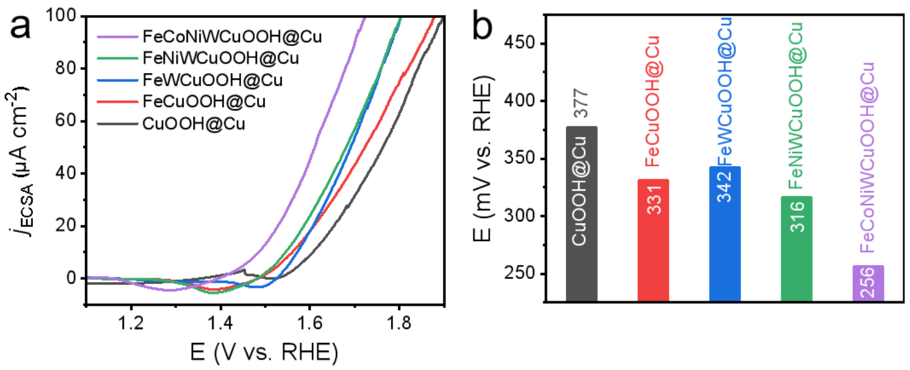

Figure S23. (a) The polarization curves normalized by ECSA and (b) corresponding overpotentials at 0.01 mA cm^{-2} of CuOOH@Cu, FeCuOOH@Cu, FeWCuOOH@Cu, FeNiWCuOOH@Cu and FeCoNiWCuOOH@Cu toward OER.

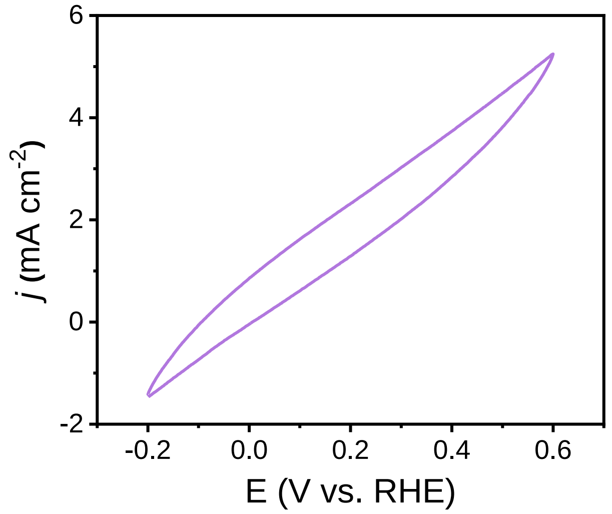

Figure S24. (a) CV curve of FeCoNiWCuOOH@Cu sample in -0.2-6 V (vs. RHE) potential interval in 1 M PBS solution.

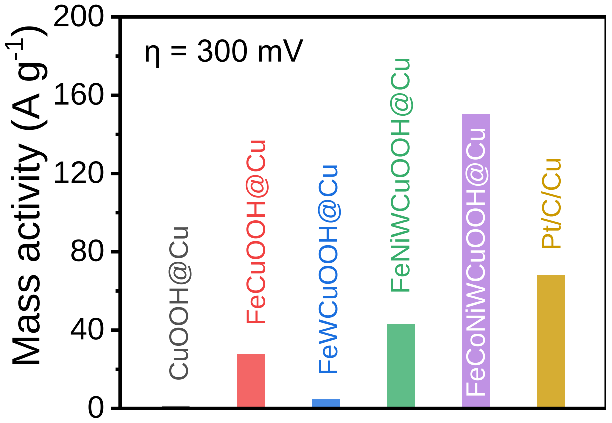

Figure S25. Mass activities of different samples in OER.

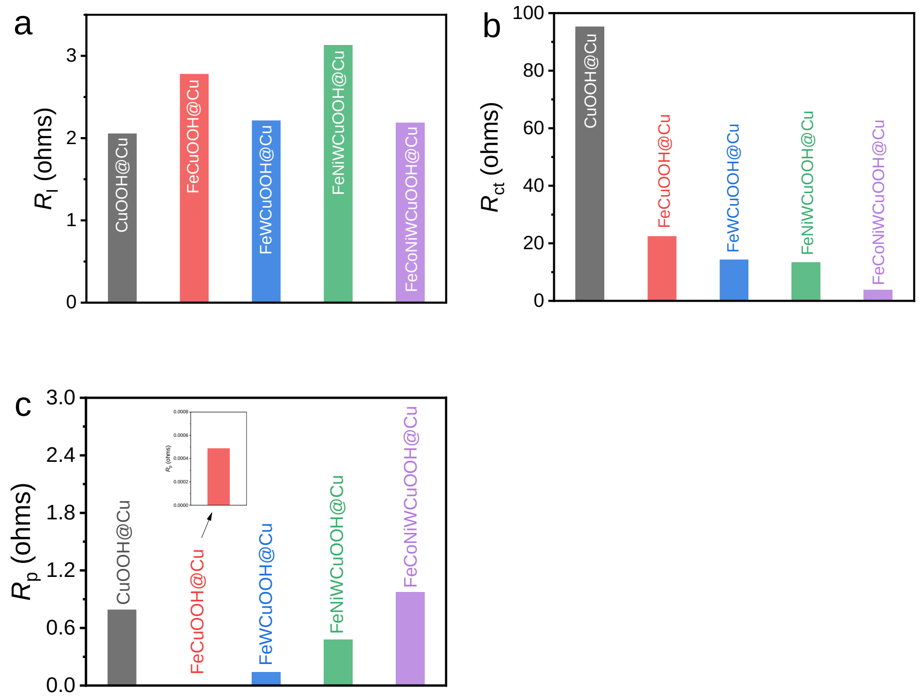

Figure S26. Comparison of (a) R_I, (b) R_{ct}, and (c) R_P for CuOOH@Cu, FeCuOOH@Cu, FeWCuOOH@Cu, FeNiWCuOOH@Cu, and FeCoNiWCuOOH@Cu electrodes according to EIS analysis.

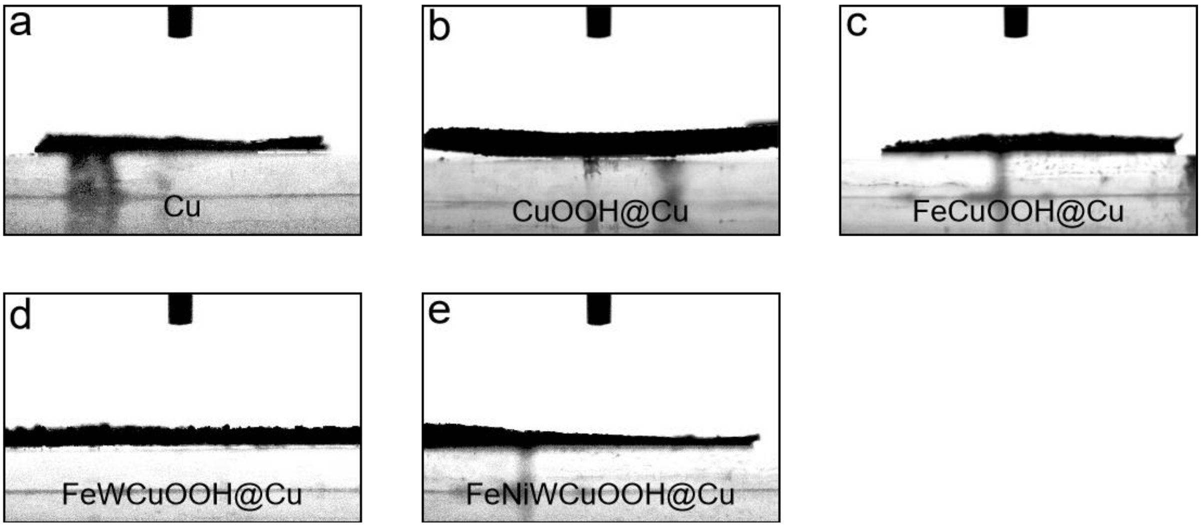

Figure S27. (a) Contact angles of Cu, CuOOH@Cu, FeCuOOH@Cu, FeWCuOOH@Cu, and FeNiWCuOOH@Cu.

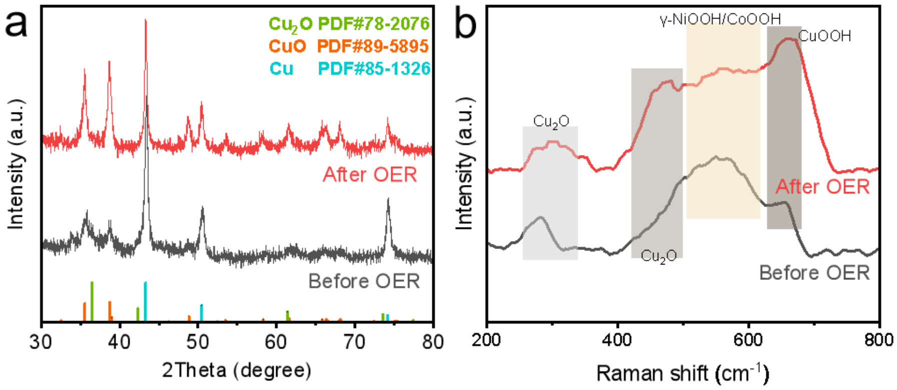

Figure S28. (a) XRD patterns and (b) Raman patterns of FeCoNiWCuOOH@Cu before and after the stability test for OER.

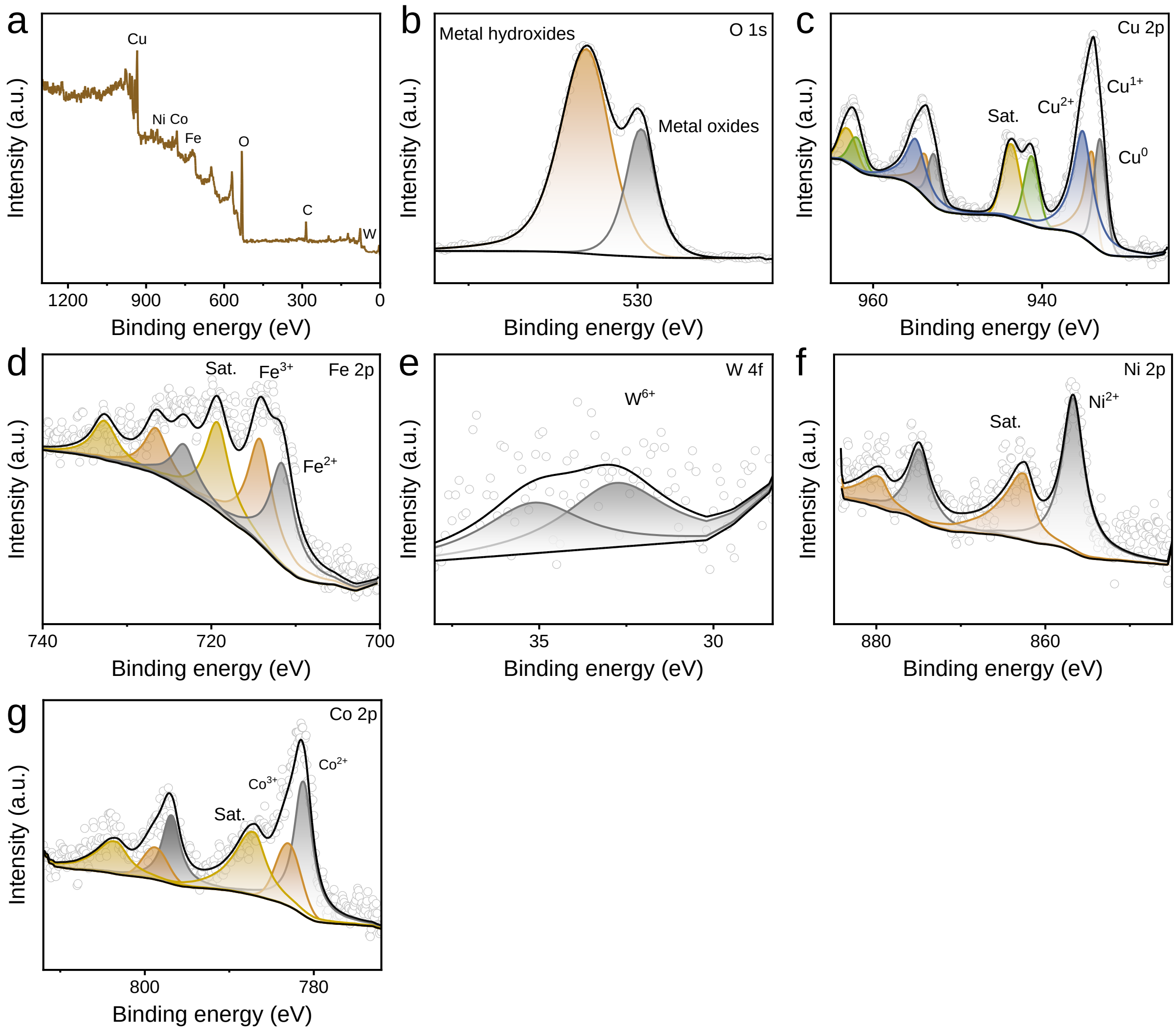

Figure S29. (a) XPS survey spectra, High-resolution (b) O 1s, (c) Cu 2p, (d) Fe 2p, (e) W 4f, (f) Ni 2p and (g) Co 2p XPS spectra of FeCoNiWCuOOH@Cu after stability test for OER.

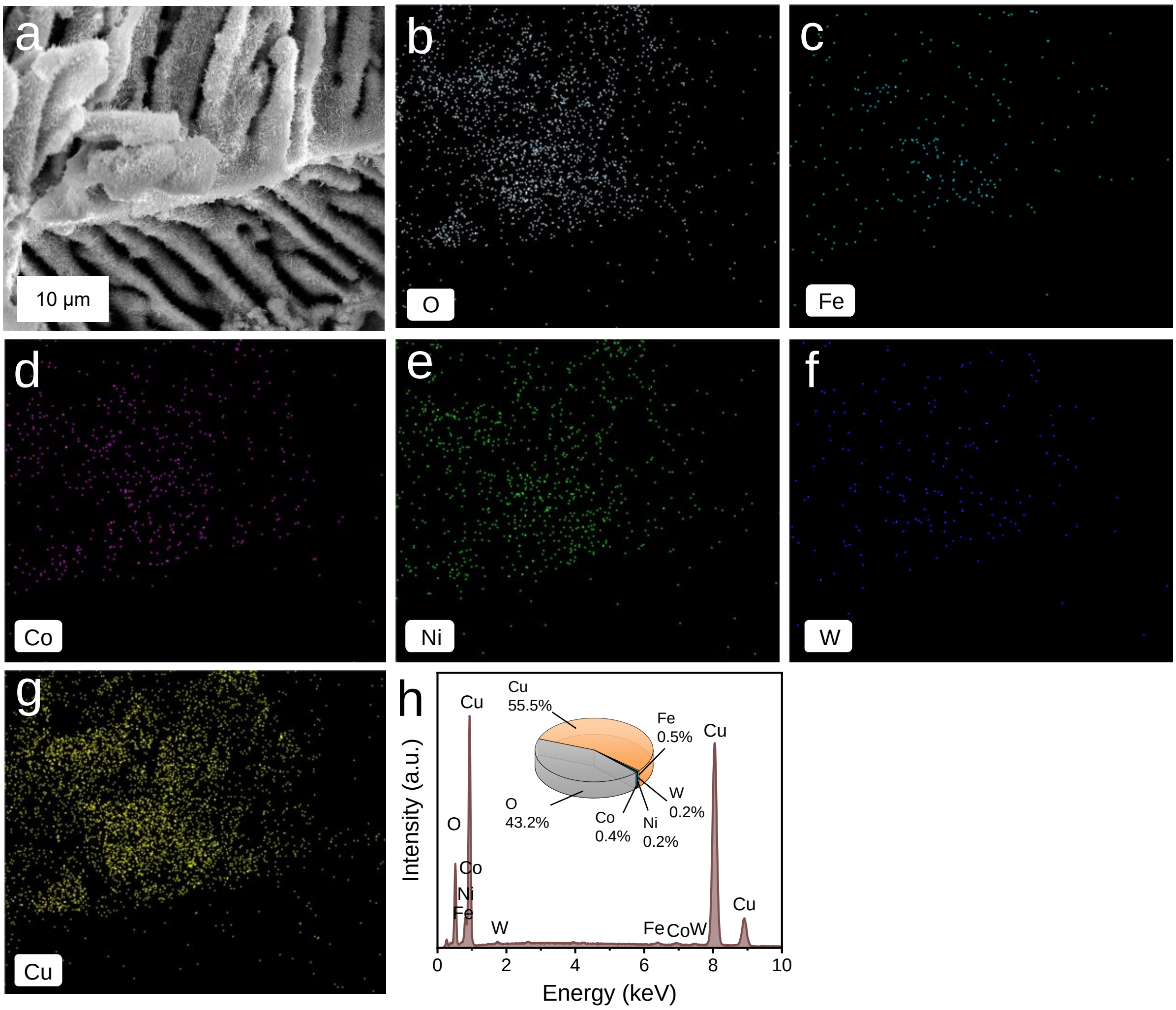

Figure S30. (a) SEM image, (b-g) corresponding EDS elemental mappings, and (h) EDS spectra of FeCoNiWCuOOH@Cu after stability test for OER. The inset of (h) is the elemental map.

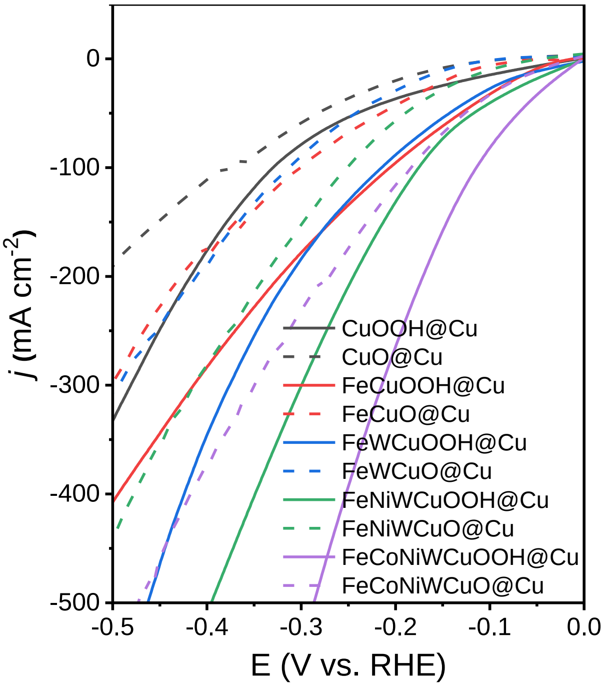

Figure S31. HER polarization curves of different samples before and after hydroxylation treatment.

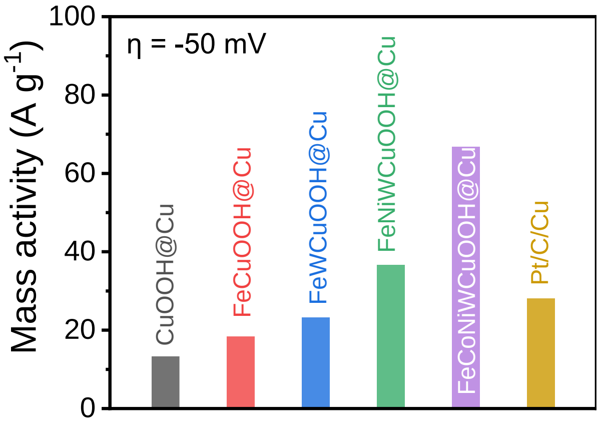

Figure S32. Mass activities of different samples in HER.

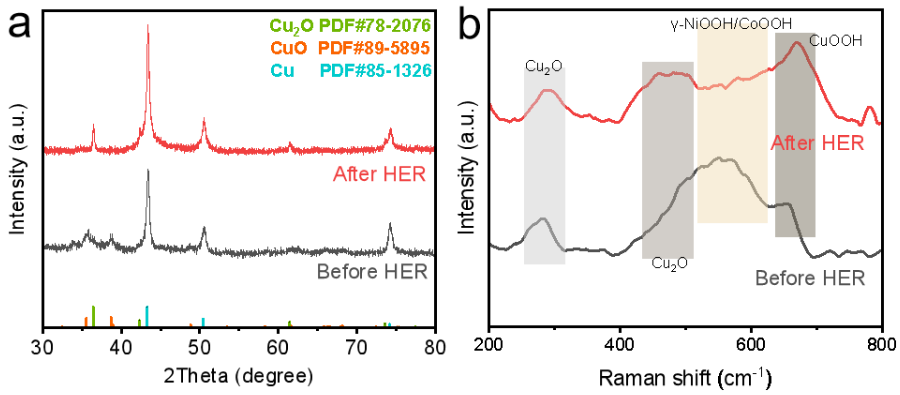

Figure S33. (a) XRD patterns and (b) Raman patterns of FeCoNiWCuOOH@Cu before and after the stability test for HER.

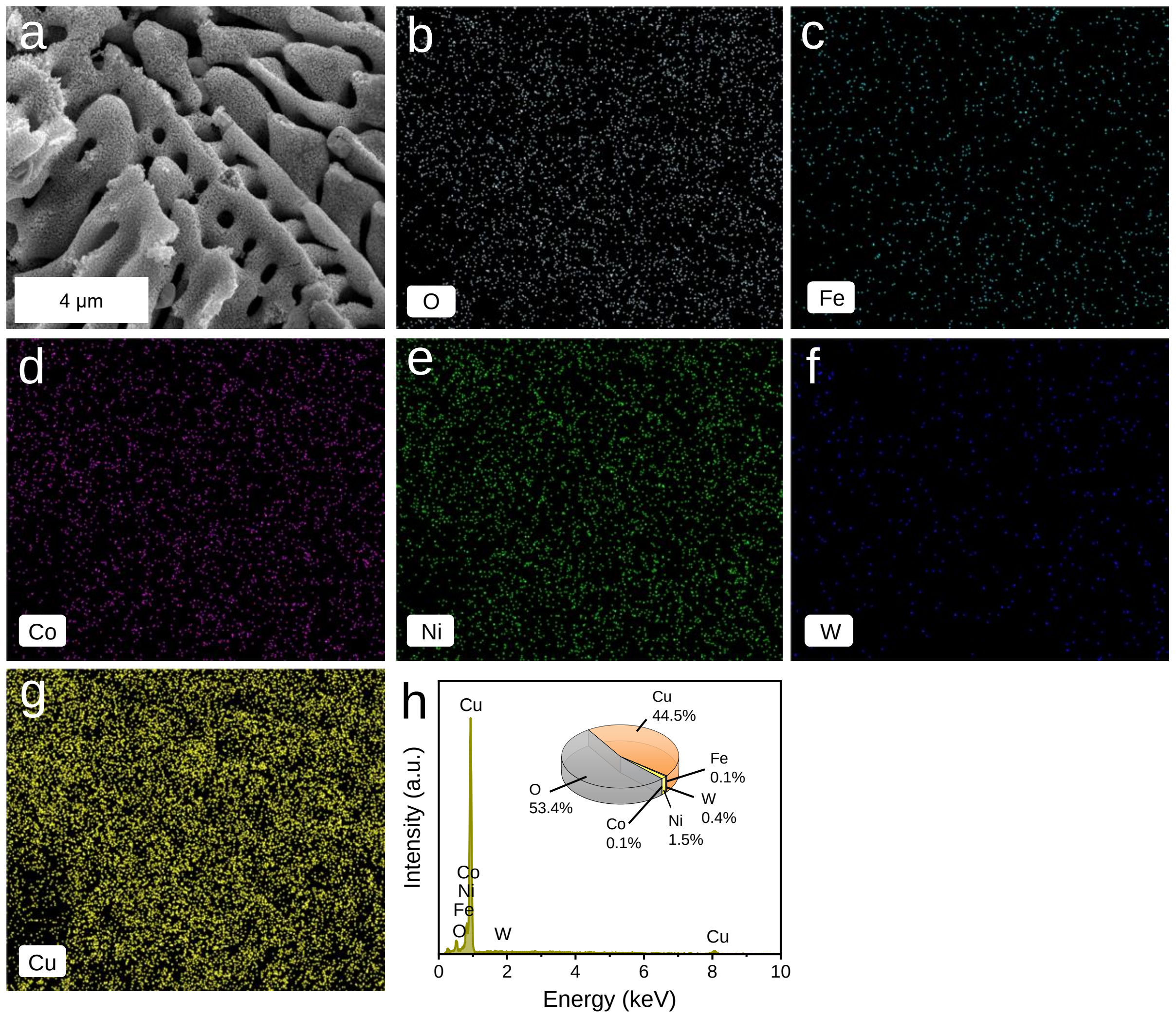

Figure S34. (a) SEM image, (b-g) corresponding EDS elemental mappings, and (h) EDS spectra of FeCoNiWCuOOH@Cu after stability test for HER. The inset of (h) is the elemental map.

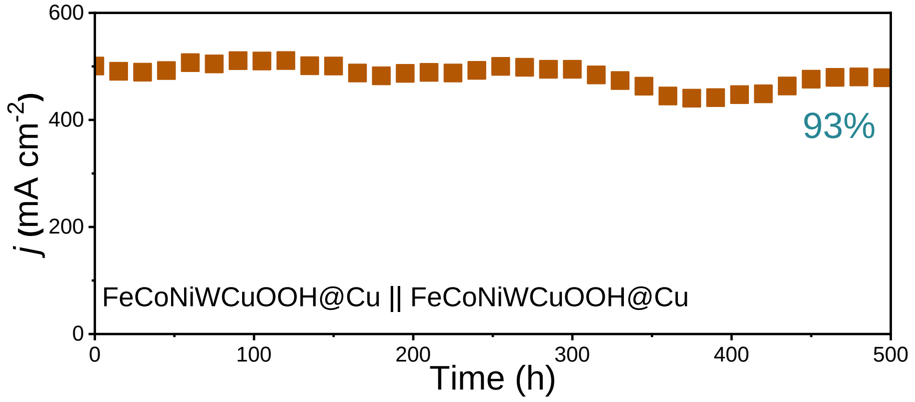

Figure S35. Constant current curve of FeCoNiWCuOOH@Cu||FeCoNiWCuOOH@Cu at 500 mA cm^{-2} for 500 h.

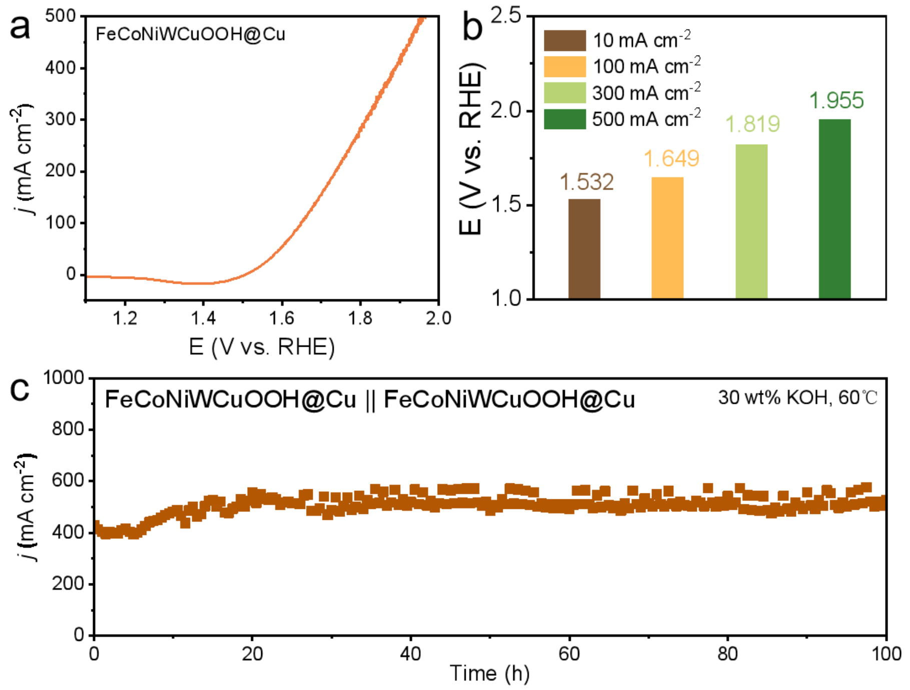

Figure S36. Constant current curve of FeCoNiWCuOOH@Cu||FeCoNiWCuOOH@Cu at 500 mA cm^{-2} for 100 h in the water electrolysis cell (30 wt% KOH, 60°C) containing an anion exchange membrane.

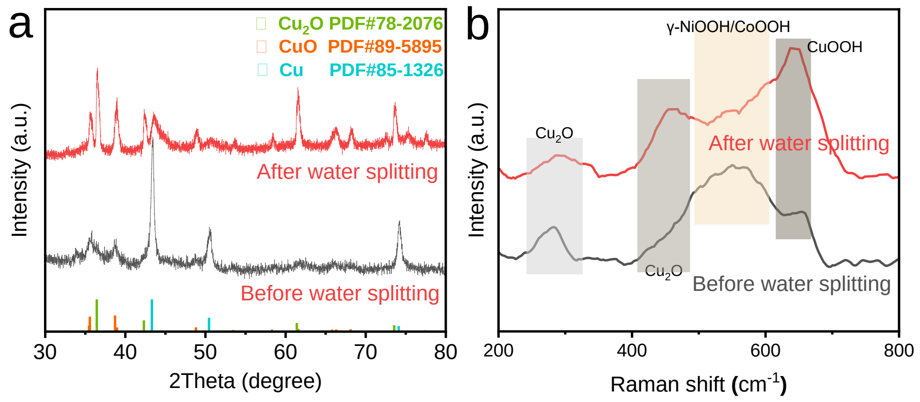

Figure S37. (a) XRD patterns and (b) Raman patterns of FeCoNiWCuOOH@Cu before and after the stability test for water splitting.

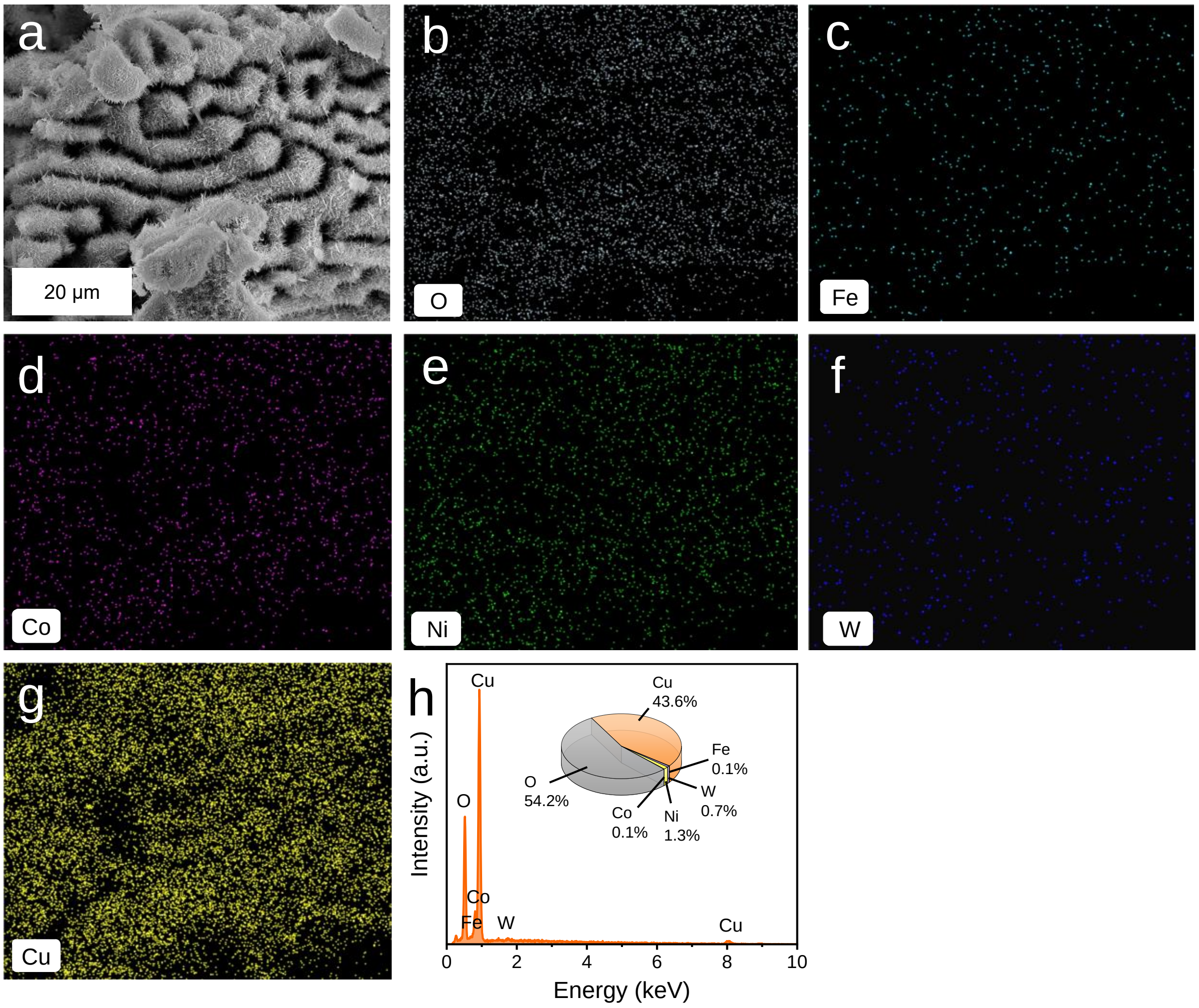

Figure S38. (a) SEM image, (b-g) corresponding EDS elemental mappings, and (h) EDS spectra of FeCoNiWCuOOH@Cu after stability test for water splitting. The inset of (h) is the elemental map.

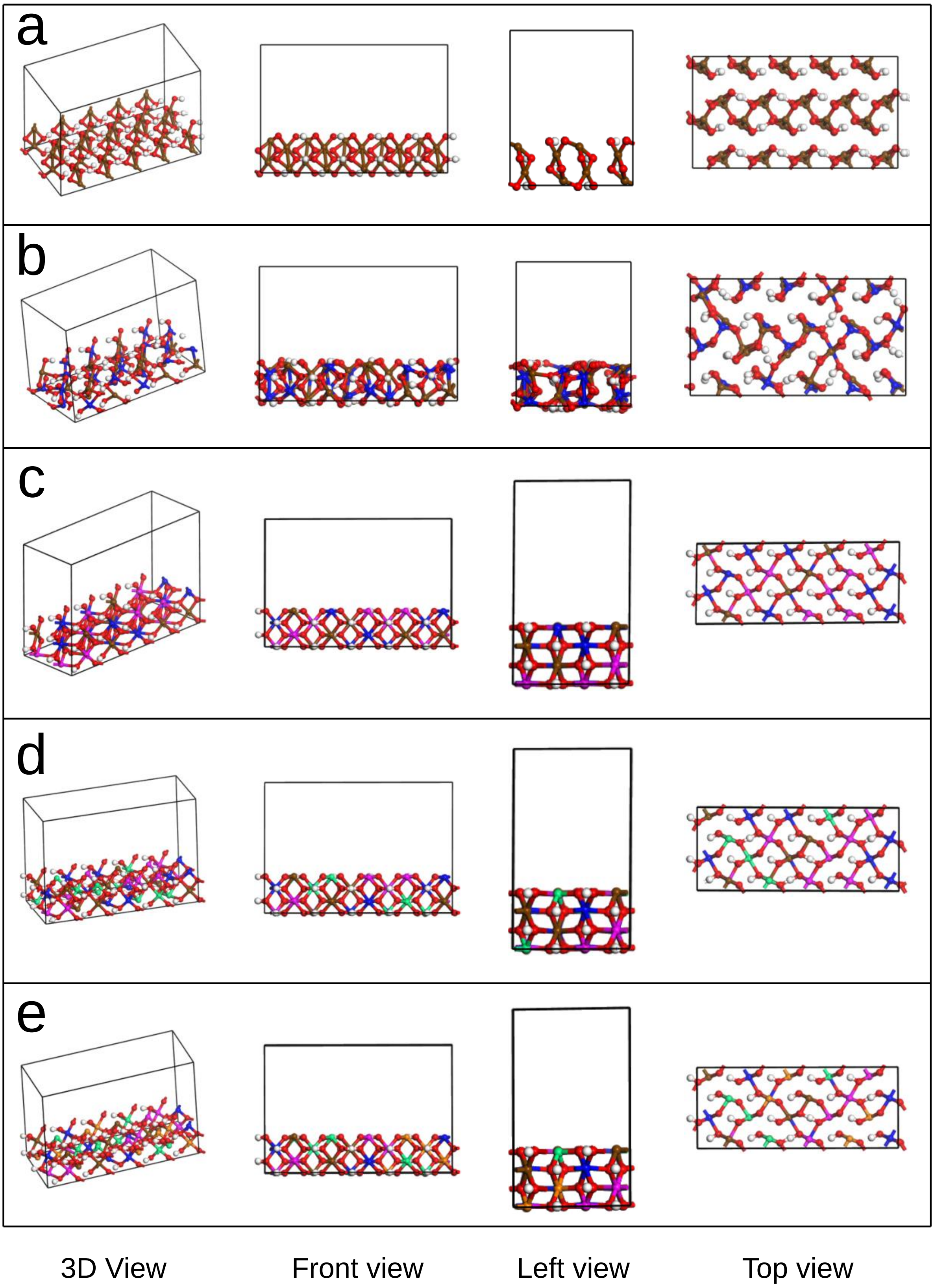

Figure S39. 3D, front, left, and top views of structure models of (a) CuOOH, (b) FeCuOOH, (c) FeWCuOOH, (d) FeNiWCuOOH, and (e) FeCoNiWCuOOH, respectively.

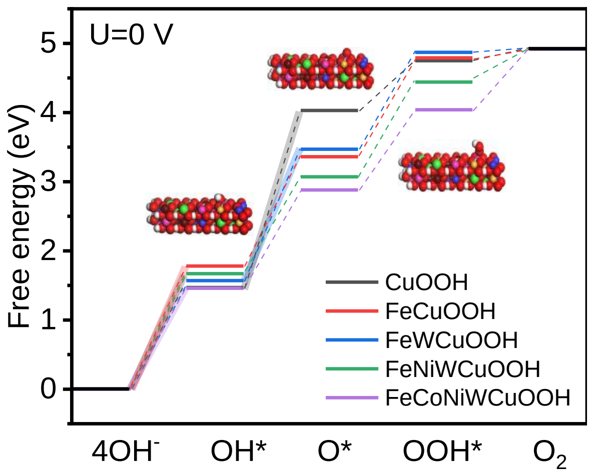

Figure S40. Free energy profiles for OER with the set potential of 0 V.

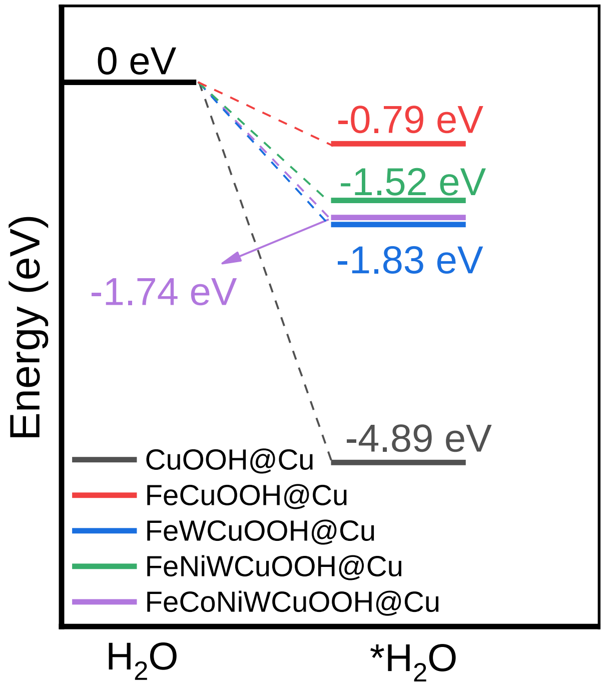

Figure S41. The adsorption energies of H_2O on different samples.

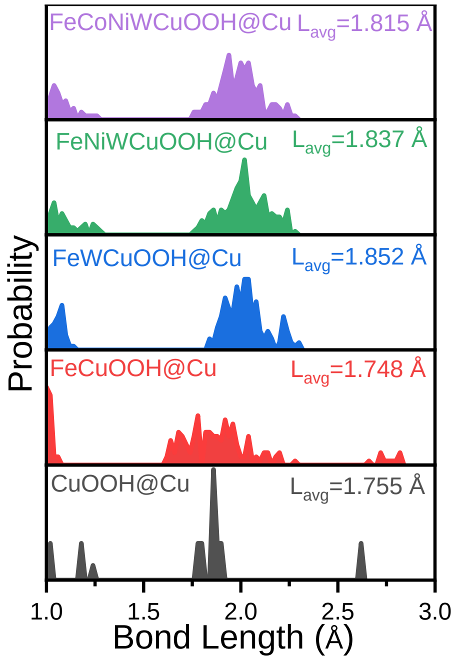

Figure S42. Comparison of the bond length distribution in different samples.

To clarify the impact of strain, we compared the bond length distributions in various high-entropy materials. For the original CuOOH@Cu catalyst, the bond lengths were mostly distributed within the range of 1.75–1.92 Å, and the distribution was relatively narrow. However, the bond length distribution range widened with the introduction of Fe, Co, Ni, and W elements, exhibiting compression/stretching strain effects. Due to changes in the average bond length (L_{avg}) (CuOOH@Cu: L_{avg}=1.755 Å; FeCuOOH@Cu: L_{avg}=1.748 Å; FeWCuOOH@Cu: L_{avg}=1.852 Å; FeNiWCuOOH@Cu: L_{avg}=1.837 Å; FeCoNiWCuOOH@Cu: L_{avg}=1.815 Å), the original catalyst experienced a trend of compression, stretching, compression, and compression. This led to changes in the occupation of antibonding orbitals, showing enhancement trends, weakening, enhancement, and enhancement. Ultimately, this adjusted ε_d to a favorable range for improving the overall water-splitting reaction rate. This also indicates that the polycrystalline boundary structures and strain effects of high-entropy materials are some of the sources leading to the high activity of HER and OER.

Table S1. The OER activities of the as-obtained FeCoNiWCuOOH@Cu electrocatalyst compared with reported related electrocatalysts (at 10 mA cm^{-2} in 1 M KOH solution).

Electrocatalysts		η (mV)	Tafel (mV dec^{-1})	Reference
**FeCoNiWCuOOH@Cu**		**200**	**24**	**This work**
Fe-based	Fe(III)-Bir	240	33	[1]
	NiFe LDH	300	40	[2]
	FeCo LDH	331	85	[3]
	P/Fe-N-C	304	65	[4]
	MIL-53(Fe)-2OH	215	45	[5]
Co-based	$CoMoO_4$	312	56	[6]
	Co_3O_4	400	49	[7]
	CoS	361	64	[8]
	CoP/rGO-400	340	66	[9]
	Co_3O_4/rm-GO	310	67	[10]
	N-CG-CoO	340	71	[11]
	CoCo-NS	353	45	[12]
	CoO_x	423	42	[13]
	Co–P FILM	345	47	[14]
	Co–P/NC	354	52	[15]
	Co_3O_4 NCs	350	101	[16]
Ni-based	m-$NiTP_yP$	267	33	[17]
	MoS_2/NiS_2	278	91	[18]
	NiS	335	89	[19]
	Ni_3N nanosheets	350	85	[20]
	Ni@NC	370	45	[21]
	NiS-Ni(OH)$_2$@aMoS$_{2+x}$	417	97	[22]
Multiple metal-based	NiFe@MoS_2	201	48	[23]
	Mo-RuCoO_x	156	69	[24]
	$Fe_{20}Co_{20}Ni_{20}Mo_{20}Al_{20}$	223	39	[25]
	AlNiCoRuMo	245	54	[26]
	CoCrFeNiMo	220	30	[27]
	FeNiCoCrMnS_2	199	39	[28]
	$(CrFeCoNi)_{97}O_3$	196	29	[29]
	FeCoNiCrMn	229	40	[30]
	FeCoNiPB	235	53	[31]
	FeCoNiMnCu	280	59	[32]
	$Fe_{0.5}CoNiCuZn_{0.8}$	340	48	[33]
Noble-metal-based	NiFeRu-LDH	290	32.4	[34]
	Ir/C-Pt/C couple	370	58.6	[34]
	IrO_2	338	47	[12]
	Ir/C	320	54	[35]

Table S2. The TOF of the as-obtained FeCoNiWCuOOH@Cu electrocatalyst for OER compared to reported related electrocatalysts (in 1 M KOH solution).

Electrocatalysts	TOF (s^{-1})	Overpotential (V)	Reference
**FeCoNiWCuOOH@Cu**	**0.764**	**1.43**	**This work**
	**2.566**	**1.48**	
	**3.652**	**1.5**	
	**5.806**	**1.53**	
	**6.579**	**1.54**	
	**7.481**	**1.55**	
	**8.442**	**1.56**	
	**12.766**	**1.6**	
KC-MLH/NF-12	0.09	1.43	[36]
	0.37	1.48	
Ru/Co_3O_{4-x}	0.056	1.5	[37]
FeCoNiMo HEA	0.051	1.53	[38]
S/N-CMF@Fe_xNi_{1-x}-MOF	0.124	1.53	[39]
m-NiTPyP/CNTs	1.16	1.53	[40]
MIL-53(Fe)-2OH	1.44	1.53	[5]
Rh-RuO_2/G	1.74	1.53	[41]
	2.39	1.53	
Co@NPC-Tfu	3.21	1.53	[42]
NiMoN/NiFe LDH	3.39	1.53	[43]
$Co_{0.5}Fe_{0.5}$-LDH	0.128	1.54	[44]
$Ir_{0.1}Ta_{0.9}O_{2.45}$	2.3	1.55	[45]
FeCoNiRu-450	0.084	1.56	[46]
Mo-$RuCoO_x$	0.176	1.6	[24]
Ir-MnO2(160)-CC	0.321	1.6	[47]

Table S3. The HER activities of the as-obtained FeCoNiWCuOOH@Cu electrocatalyst compared with reported related electrocatalysts (at 10 mA cm^{-2} in 1 M KOH solution).

Electrocatalysts		η (mV)	Tafel (mV dec^{-1})	Reference
**FeCoNiWCuOOH@Cu**		**16**	**14**	**This work**
Fe-based	FeS_2	96	78	[48]
	B-Fe_7S_8/FeS_2	113	57	[49]
	Fe–CoP/Ti	78	75	[50]
	FeS_2 NSs	186	123	[51]
Co-based	c-$CoSe_2$/CC	190	85	[52]
	Co/CoP-5	253	73.8	[53]
	Co_9S_8@NOSCb	320	105	[54]
	CoO_x@CN	232	115	[55]
	HNDCM-Co/CoP	138	64	[56]
	Cr-Co_xP	100	75	[57]
	Fe@Co_9S_8	44	66	[58]
Ni-based	Ni_3N@CQDs	69	108	[59]
	Ni/Ni_xP_y	130	58.5	[60]
	Ni_3S_2/MoS_2	110	55	[61]
	NF-NiS_2	67	63	[62]
	Co-Ni_3S_2@CNT/GNF	155	138	[63]
	Mo-Ni_3S_2	212	98	[64]
	N-Ni_3S_2	155	113	[65]
	m-$NiTP_yP$	138	83	[17]
Multiple metal-based	FeCoNi	64	125	[66]
	$NiFeO_x$@NiCu	70	68	[67]
	NiZn-CoO	53	47	[68]
	Ni_4Mo/MoO_x/Cu	16	64	[69]
	NiFeSe	50	49	[70]
	NiCoWS	70	112	[71]
	H-Fe-CoMoS	137	98	[72]
	$Fe_{0.54}Co_{0.46}S_{0.92}$/CNTs/C C	70	64	[73]
	NiFe@MoS_2	67	42	[23]
Noble-metal-based	β-$Ni(OH)_2$/Pt	92	51	[74]
	Sr_2RuO_4	61	50	[75]
	IrP_2@NC	28	43	[76]
	CoNiPt NFs	25	48	[77]
	$Cu_{2-x}S$@Ru	82	29	[78]
	Ru/NC	24	24	[79]
	Mo-$RuCoO_x$	41	42	[24]

Table S4. The TOF of the as-obtained FeCoNiWCuOOH@Cu electrocatalyst for HER compared to reported related electrocatalysts (in 1 M KOH solution).

Electrocatalysts	TOF (s^{-1})	Overpotential (V)	Reference
**FeCoNiWCuOOH@Cu**	**2.34**	**0.025**	**This work**
	**5.166**	**0.05**	
	**8.573**	**0.075**	
	**12.766**	**0.1**	
	**19.223**	**0.13**	
	**41.292**	**0.2**	
Ru@C_2N	0.75	0.025	[80]
PtPdRhRuCu	4.5	0.05	[81]
W-ACs	0.12	0.05	[82]
FeCoNiCuPtIr	0.311	0.05	[83]
Rh_{15}/NSC	0.143	0.075	[84]
Ni_2P	0.015	0.1	[85]
Ni_4Mo/GNS	0.11	0.1	[86]
FeCoNiRu-450	0.046	0.1	[46]
B-Os aerogels	1.17	0.1	[87]
W-$NiS_{0.5}Se_{0.5}$	0.21	0.13	[88]
m-NiTPyP/CNTs	0.45	0.2	[40]
W_2N/WC	0.21	0.2	[89]

Table S5. The water splitting activities of the as-obtained FeCoNiWCuOOH@Cu electrocatalyst compared with reported related electrocatalysts (in 1 M KOH solution).

Electrocatalysts	η (V@10 mA cm^{-2})	Stability time (h)	Reference
**FeCoNiWCuOOH@Cu**	**1.40**	**1 000 (300 mA cm^{-2})**<br>**500 (500 mA cm^{-2})**	**This work**
Ru_1/D-NiFe LDH	1.44	100 (100 mA cm^{-2})	[90]
$Ni_{cluster}$-Ru NWs	1.442	24 (5 mA cm^{-2})	[91]
Rh/NiFeRh-LDH/NF	1.46	10 000 s<br>(20 mA cm^{-2})	[92]
Ru-NiFe-P/NF	1.47	20 (10 mA cm^{-2})	[93]
(Ru-Ni)O_x	1.48	10 (50 mA cm^{-2})	[94]
NiTe@NiFe-LDH‖NiTe@RuO_2	1.49	27 (10 mA cm^{-2})	[95]
NiVIr LDH	1.49	15 (10 mA cm^{-2})	[96]
DH-CuCo-P@NC/CC	1.494	12 (20 mA cm^{-2})	[97]
Cr-doped FeNi-P/NCN	1.5	20 (10 mA cm^{-2})	[98]
Rh SAC-CuO NAs/CF	1.51	25 (10 mA cm^{-2})	[99]
Ru-NiCoP/NF	1.515	50 (60 mA cm^{-2})	[100]
NiFeRu-LDH/NF	1.52	10 (10 mA cm^{-2})	[34]
NF@Fe_2-Ni_2P/C	1.53	22 (300 mA cm^{-2})	[101]
Pt/$LiCoO_2$	1.54	24 (50 mA cm^{-2})	[102]
V-doped CoP@CeO_X	1.56	35 (20 mA cm^{-2})	[103]
Ni_2P-Fe_2P/NF	1.561	48 (100 mA cm^{-2})	[104]
$Ni_{1.5}Co_{0.5}$@N-C NT/NFs	1.57	27 (10 mA cm^{-2})	[105]
CoP-InNC@CNT	1.58	10 (15 mA cm^{-2})	[106]
CoP/NCNHP	1.64	26 (20 mA cm^{-2})	[107]
Co/NBC-900	1.68	6 (10 mA cm^{-2})	[108]

Table S6. Water adsorption energy on the surfaces of electrocatalysts.

Samples	Adsorption site	Structural model	Free energy (eV)
CuOOH	Cu	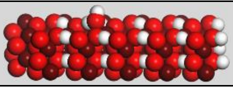	-4.89
FeCuOOH	Cu	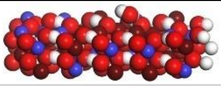	-0.79
	Fe	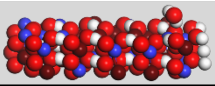	-0.57
FeWCuOOH	Cu	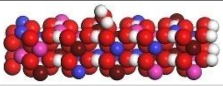	-0.86
	Fe	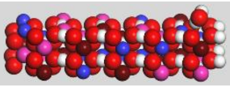	-0.76
	W	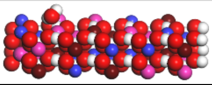	-1.83
FeNiWCuOOH	Cu	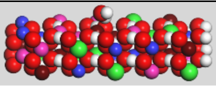	-1.01
	Fe	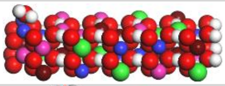	-1.04
	W	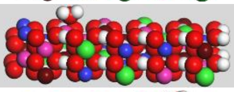	-1.52
	Ni	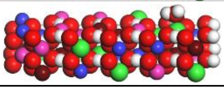	-1.13
FeCoNiWCuOOH	Cu	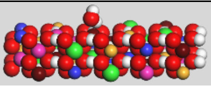	-0.82
	Fe	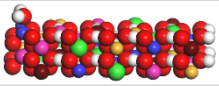	-1.43
	W	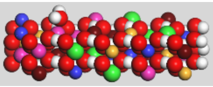	-1.74
	Ni	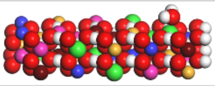	-1.12
	Co	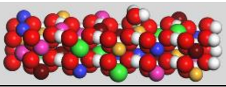	-1.25

Note: The structural model represents the most stable configurations of each adsorbed intermediate. Blue, pink, cyan, yellow, purple, red, and white spheres represent Fe, Co, Ni, W, Cu, O, and H elements, respectively.

References

[1] M. Ju, Z. W. Chen, H. Zhu, R. M. Cai, Z. D. Lin, Y. P. Chen, Y. J. Wang, J. L. Gao, X. Long, S. H. Yang, *J. Am. Chem. Soc.* **2023**, *145*, 11215.

[2] D. Friebel, M. W. Louie, M. Bajdich, K. E. Sanwald, Y. Cai, A. M. Wise, M.-J. Cheng, D. Sokaras, T.-C. Weng, R. Alonso-Mori, *J. Am. Chem. Soc.* **2015**, *137*, 1305.

[3] B. Zhang, X. Zheng, O. Voznyy, R. Comin, M. Bajdich, M. García-Melchor, L. Han, J. Xu, M. Liu, L. Zheng, *Science* **2016**, *352*, 333.

[4] Y. Zhou, R. Lu, X. Tao, Z. Qiu, G. Chen, J. Yang, Y. Zhao, X. Feng, K. Müllen, *J. Am. Chem. Soc.* **2023**, *145*, 3647.

[5] C. Zhang, Q. Qi, Y. Mei, J. Hu, M. Sun, Y. Zhang, B. Huang, L. Zhang, S. Yang, *Adv. Mater.* **2023**, *35*, 2208904.

[6] M. Q. Yu, L. X. Jiang, H. G. Yang, *Chem. Commun.* **2015**, *51*, 14361.

[7] J. A. Koza, Z. He, A. S. Miller, J. A. Switzer, *Chem. Mater.* **2012**, *24*, 3567.

[8] T. Liu, Y. Liang, Q. Liu, X. Sun, Y. He, A. M. Asiri, *Electrochem. Commun.* **2015**, *60*, 92.

[9] L. Jiao, Y.-X. Zhou, H.-L. Jiang, *Chem. Sci.* **2016**, *7*, 1690.

[10] Y. Liang, Y. Li, H. Wang, J. Zhou, J. Wang, T. Regier, H. Dai, *Nat. Mater.* **2011**, *10*, 780.

[11] S. Mao, Z. Wen, T. Huang, Y. Hou, J. Chen, *Energy Environ. Sci.* **2014**, *7*, 609.

[12] F. Song, X. Hu, *Nat. Commun.* **2014**, *5*, 4477.

[13] L. Trotochaud, J. K. Ranney, K. N. Williams, S. W. Boettcher, *J. Am. Chem. Soc.* **2012**, *134*, 17253.

[14] N. Jiang, B. You, M. Sheng, Y. Sun, *Angew. Chem.* **2015**, *127*, 6349.

[15] B. You, N. Jiang, M. Sheng, S. Gul, J. Yano, Y. Sun, *Chem. Mater.* **2015**, *27*, 7636.

[16] S. Du, Z. Ren, J. Zhang, J. Wu, W. Xi, J. Zhu, H. Fu, *Chem. Commun.* **2015**, *51*, 8066.

[17] Y. Y. Zhang, S. T. Chen, Y. X. Zhang, R. J. Li, B. Zhao, T. Y. Peng, *Adv. Mater.* **2023**, *35*, 2210727.

[18] J. Lin, P. Wang, H. Wang, C. Li, X. Si, J. Qi, J. Cao, Z. Zhong, W. Fei, J. Feng, *Adv. Sci.* **2019**, *6*, 1900246.

[19] W. Zhu, X. Yue, W. Zhang, S. Yu, Y. Zhang, J. Wang, J. Wang, *Chem. Commun.* **2016**,

52, 1486.

[20] K. Xu, P. Chen, X. Li, Y. Tong, H. Ding, X. Wu, W. Chu, Z. Peng, C. Wu, Y. Xie, *J. Am. Chem. Soc.* **2015**, *137*, 4119.

[21] Y. Xu, W. Tu, B. Zhang, S. Yin, Y. Huang, M. Kraft, R. Xu, *Adv. Mater.* **2017**, *29*, 1605957.

[22] T. Yoon, K. S. Kim, *Adv. Funct. Mater.* **2016**, *26*, 7386.

[23] Z. Jiang, W. Zhou, C. Hu, X. Luo, W. Zeng, X. Gong, Y. Yang, T. Yu, W. Lei, C. Yuan, *Adv. Mater.* **2023**, *35*, 2300505.

[24] Y. Zhang, R. Lu, C. Wang, Y. Zhao, L. Qi, *Adv. Funct. Mater.* **2023**, *33*, 2303073.

[25] Y. F. Cui, S. D. Jiang, Q. Fu, R. Wang, P. Xu, Y. Sui, X. J. Wang, Z. L. Ning, J. F. Sun, X. Sun, *Adv. Funct. Mater.* **2023**, 2306889.

[26] Z. Jin, J. Lyu, Y.-L. Zhao, H. Li, X. Lin, G. Xie, X. Liu, J.-J. Kai, H.-J. Qiu, *ACS Materials Lett.* **2020**, *2*, 1698.

[27] J. Tang, J. Xu, Z. Ye, Y. Ma, X. Li, J. Luo, Y. Huang, *J. Alloys Compd.* **2021**, *885*, 160995.

[28] T. X. Nguyen, Y. H. Su, C. C. Lin, J. M. Ting, *Adv. Funct. Mater.* **2021**, *31*, 2106229.

[29] Z. J. Chen, T. Zhang, X. Y. Gao, Y. J. Huang, X. H. Qin, Y. F. Wang, K. Zhao, X. Peng, C. Zhang, L. Liu, *Adv. Mater.* **2021**, *33*, 2101845.

[30] T. X. Nguyen, Y. H. Su, C. C. Lin, J. Ruan, J. M. Ting, *Adv. Sci.* **2021**, *8*, 2002446.

[31] Q. Wang, J. Li, Y. Li, G. Shao, Z. Jia, B. Shen, *Nano Res.* **2022**, *15*, 8751.

[32] K. Huang, D. Peng, Z. Yao, J. Xia, B. Zhang, H. Liu, Z. Chen, F. Wu, J. Wu, Y. Huang, *Chem. Eng. J.* **2021**, *425*, 131533.

[33] J. Huang, P. Wang, P. Li, H. Yin, D. Wang, *J. Mater. Sci. Technol.* **2021**, *93*, 110.

[34] G. Chen, T. Wang, J. Zhang, P. Liu, H. Sun, X. Zhuang, M. Chen, X. Feng, *Adv. Mater.* **2018**, *30*, 1706279.

[35] H. B. Yang, J. Miao, S.-F. Hung, J. Chen, H. B. Tao, X. Wang, L. Zhang, R. Chen, J. Gao, H. M. Chen, *Sci. Adv.* **2016**, *2*, e1501122.

[36] G. Mu, G. Wang, Q. Huang, Y. Miao, D. Wen, D. Lin, C. Xu, Y. Wan, F. Xie, W. Guo, *Adv. Funct. Mater.* **2023**, *33*, 2211260.

[37] C.-Z. Yuan, S. Wang, K. San Hui, K. Wang, J. Li, H. Gao, C. Zha, X. Zhang, D. A. Dinh,

X.-L. Wu, *ACS Catal.* **2023**, *13*, 2462.

[38] Y. J. Mei, Y. B. Feng, C. X. Zhang, Y. Zhang, Q. L. Qi, J. Hu, *ACS Catal.* **2022**, *12*, 10808.

[39] Y. Zhao, X. F. Lu, Z. P. Wu, Z. Pei, D. Luan, X. W. Lou, *Adv. Mater.* **2023**, *35*, 2207888.

[40] Y. Zhang, S. Chen, Y. Zhang, R. Li, B. Zhao, T. Peng, *Adv. Mater.* **2023**, *35*, 2210727.

[41] Y. Wang, R. Yang, Y. Ding, B. Zhang, H. Li, B. Bai, M. Li, Y. Cui, J. Xiao, Z.-S. Wu, *Nat. Commun.* **2023**, *14*, 1412.

[42] J. Zhang, R. Z. Sun, X. F. Zhang, J. X. Wu, Y. H. Dou, X. Y. Zhu, L. H. Yu, L. Y. Guo, M. L. Liu, L. Guo, L. M. Cao, C. T. He, X. M. Chen, *Adv. Funct. Mater.* **2022**, *32*, 2202119.

[43] P. Zhai, C. Wang, Y. Zhao, Y. Zhang, J. Gao, L. Sun, J. Hou, *Nat. Commun.* **2023**, *14*, 1873.

[44] S. Shankar Naik, J. Theerthagiri, F. S. Nogueira, S. J. Lee, A. Min, G.-A. Kim, G. Maia, L. M. Pinto, M. Y. Choi, *ACS Catal.* **2023**, *13*, 1477.

[45] S. Wang, T. Shen, C. Yang, G. Luo, D. Wang, *ACS Catal.* **2023**, *13*, 8670.

[46] K. Huang, J. Xia, Y. Lu, B. Zhang, W. Shi, X. Cao, X. Zhang, L. M. Woods, C. Han, C. Chen, *Adv. Sci.* **2023**, *10*, 2300094.

[47] Y. Weng, K. Wang, S. Li, Y. Wang, L. Lei, L. Zhuang, Z. Xu, *Adv. Sci.* **2023**, *10*, 2205920.

[48] R. Miao, B. Dutta, S. Sahoo, J. He, W. Zhong, S. A. Cetegen, T. Jiang, S. P. Alpay, S. L. Suib, *J. Am. Chem. Soc.* **2017**, *139*, 13604.

[49] J. Wu, Q. Zhang, K. Shen, R. Zhao, W. Zhong, C. Yang, H. Xiang, X. Li, N. Yang, *Adv. Funct. Mater.* **2022**, *32*, 2107802.

[50] C. Tang, R. Zhang, W. Lu, L. He, X. Jiang, A. M. Asiri, X. Sun, *Adv. Mater.* **2017**, *29*, 1602441.

[51] Y. Li, J. Yin, L. An, M. Lu, K. Sun, Y. Q. Zhao, D. Gao, F. Cheng, P. Xi, *Small* **2018**, *14*, 1801070.

[52] P. Chen, K. Xu, S. Tao, T. Zhou, Y. Tong, H. Ding, L. Zhang, W. Chu, C. Wu, Y. Xie, *Adv. Mater.* **2016**, *28*, 7527.

[53] Z. H. Xue, H. Su, Q. Y. Yu, B. Zhang, H. H. Wang, X. H. Li, J. S. Chen, *Adv. Energy*

Mater. **2017**, *7*, 1602355.

[54] S. Huang, Y. Meng, S. He, A. Goswami, Q. Wu, J. Li, S. Tong, T. Asefa, M. Wu, *Adv. Funct. Mater.* **2017**, *27*, 1606585.

[55] H. Jin, J. Wang, D. Su, Z. Wei, Z. Pang, Y. Wang, *J. Am. Chem. Soc.* **2015**, *137*, 2688.

[56] H. Wang, S. Min, Q. Wang, D. Li, G. Casillas, C. Ma, Y. Li, Z. Liu, L.-J. Li, J. Yuan, *ACS Nano* **2017**, *11*, 4358.

[57] Y. Song, M. Sun, S. Zhang, X. Zhang, P. Yi, J. Liu, B. Huang, M. Huang, L. Zhang, *Adv. Funct. Mater.* **2023**, *33*, 2214081.

[58] B. Tian, L. G. Sun, D. R. Ho, *Adv. Funct. Mater.* **2023**, *33*, 2210298.

[59] M. Zhou, Q. Weng, Z. I. Popov, Y. Yang, L. Y. Antipina, P. B. Sorokin, X. Wang, Y. Bando, D. Golberg, *ACS Nano* **2018**, *12*, 4148.

[60] G. F. Chen, T. Y. Ma, Z. Q. Liu, N. Li, Y. Z. Su, K. Davey, S. Z. Qiao, *Adv. Funct. Mater.* **2016**, *26*, 3314.

[61] J. Zhang, T. Wang, D. Pohl, B. Rellinghaus, R. Dong, S. Liu, X. Zhuang, X. Feng, *Angew. Chem.* **2016**, *128*, 6814.

[62] Q. Ma, C. Hu, K. Liu, S.-F. Hung, D. Ou, H. M. Chen, G. Fu, N. Zheng, *Nano Energy* **2017**, *41*, 148.

[63] F. Wang, Y. Zhu, W. Tian, X. Lv, H. Zhang, Z. Hu, Y. Zhang, J. Ji, W. Jiang, *J. Mater. Chem. A* **2018**, *6*, 10490.

[64] C. Wu, B. Liu, J. Wang, Y. Su, H. Yan, C. Ng, C. Li, J. Wei, *Appl. Surf. Sci.* **2018**, *441*, 1024.

[65] T. Kou, T. Smart, B. Yao, I. Chen, D. Thota, Y. Ping, Y. Li, *Adv. Energy Mater.* **2018**, *8*, 1703538.

[66] Q. Zhang, N. M. Bedford, J. Pan, X. Lu, R. Amal, *Adv. Energy Mater.* **2019**, *9*, 1901312.

[67] Y. Zhou, Z. Wang, Z. Pan, L. Liu, J. Xi, X. Luo, Y. Shen, *Adv. Mater.* **2019**, *31*, 1806769.

[68] T. Ling, T. Zhang, B. Ge, L. Han, L. Zheng, F. Lin, Z. Xu, W. B. Hu, X. W. Du, K. Davey, *Adv. Mater.* **2019**, *31*, 1807771.

[69] Y. An, X. Long, M. Ma, J. Hu, H. Lin, D. Zhou, Z. Xing, B. Huang, S. Yang, *Adv. Energy Mater.* **2019**, *9*, 1901454.

[70] G. Yilmaz, C. F. Tan, Y. F. Lim, G. W. Ho, *Adv. Energy Mater.* **2019**, *9*, 1802983.

[71] M. Ma, J. Xu, H. Wang, X. Zhang, S. Hu, W. Zhou, H. Liu, *Appl. Catal. B-Environ.* **2021**, *297*, 120455.

[72] Y. Guo, X. Zhou, J. Tang, S. Tanaka, Y. V. Kaneti, J. Na, B. Jiang, Y. Yamauchi, Y. Bando, Y. Sugahara, *Nano Energy* **2020**, *75*, 104913.

[73] W. Xiong, Z. Guo, H. Li, R. Zhao, X. Wang, *ACS Energy Lett.* **2017**, *2*, 2778.

[74] X. Yu, J. Zhao, L.-R. Zheng, Y. Tong, M. Zhang, G. Xu, C. Li, J. Ma, G. Shi, *ACS Energy Lett.* **2017**, *3*, 237.

[75] Y. Zhu, H. A. Tahini, Z. Hu, J. Dai, Y. Chen, H. Sun, W. Zhou, M. Liu, S. C. Smith, H. Wang, *Nat. Commun.* **2019**, *10*, 149.

[76] Z. Pu, J. Zhao, I. S. Amiinu, W. Li, M. Wang, D. He, S. Mu, *Energy Environ. Sci.* **2019**, *12*, 952.

[77] Y. D. Pan, J. K. Gao, E. J. Lv, T. T. Li, H. Xu, L. Sun, A. Nairan, Q. C. Zhang, *Adv. Funct. Mater.* **2023**, *33*, 2303833.

[78] D. Yoon, J. Lee, B. Seo, B. Kim, H. Baik, S. H. Joo, K. Lee, *Small* **2017**, *13*, 1700052.

[79] Y. Zhu, K. Fan, C. S. Hsu, G. Chen, C. Chen, T. Liu, Z. Lin, S. She, L. Li, H. Zhou, *Adv. Mater.* **2023**, *35*, 2301133.

[80] J. Mahmood, F. Li, S.-M. Jung, M. S. Okyay, I. Ahmad, S.-J. Kim, N. Park, H. Y. Jeong, J.-B. Baek, *Nat. Nanotechnol.* **2017**, *12*, 441.

[81] Y. Kang, O. Cretu, J. Kikkawa, K. Kimoto, H. Nara, A. S. Nugraha, H. Kawamoto, M. Eguchi, T. Liao, Z. Sun, *Nat. Commun.* **2023**, *14*, 4182.

[82] Z. Chen, Y. Xu, D. Ding, G. Song, X. Gan, H. Li, W. Wei, J. Chen, Z. Li, Z. Gong, *Nat. Commun.* **2022**, *13*, 763.

[83] Y. Lu, K. Huang, X. Cao, L. Zhang, T. Wang, D. Peng, B. Zhang, Z. Liu, J. Wu, Y. Zhang, *Adv. Funct. Mater.* **2022**, *32*, 2110645.

[84] X. Bu, Y. Bu, Q. Quan, S. Yang, Y. Meng, D. Chen, Z. Lai, P. Xie, D. Yin, D. Li, *Adv. Funct. Mater.* **2022**, *32*, 2206006.

[85] E. J. Popczun, J. R. McKone, C. G. Read, A. J. Biacchi, A. M. Wiltrout, N. S. Lewis, R. E. Schaak, *J. Am. Chem. Soc.* **2013**, *135*, 9267.

[86] Y. Zhou, T. Lin, X. Luo, Z. Yan, J. Wu, J. Wang, Y. Shen, *J. Catal.* **2020**, *388*, 122.

[87] Y. Li, C.-K. Peng, H. Hu, S.-Y. Chen, J.-H. Choi, Y.-G. Lin, J.-M. Lee, *Nat. Commun.* **2022**, *13*, 1143.

[88] Y. Wang, X. Li, M. Zhang, J. Zhang, Z. Chen, X. Zheng, Z. Tian, N. Zhao, X. Han, K. Zaghib, *Adv. Mater.* **2022**, *34*, 2107053.

[89] J. Diao, Y. Qiu, S. Liu, W. Wang, K. Chen, H. Li, W. Yuan, Y. Qu, X. Guo, *Adv. Mater.* **2020**, *32*, 1905679.

[90] P. Zhai, M. Xia, Y. Wu, G. Zhang, J. Gao, B. Zhang, S. Cao, Y. Zhang, Z. Li, Z. Fan, *Nat. Commun.* **2021**, *12*, 4587.

[91] T. Zhu, S. Liu, B. Huang, Q. Shao, M. Wang, F. Li, X. Tan, Y. Pi, S.-C. Weng, B. Huang, *Energy Environ. Sci.* **2021**, *14*, 3194.

[92] B. Zhang, C. Zhu, Z. Wu, E. Stavitski, Y. H. Lui, T.-H. Kim, H. Liu, L. Huang, X. Luan, L. Zhou, *Nano Lett.* **2019**, *20*, 136.

[93] M. Qu, Y. Jiang, M. Yang, S. Liu, Q. Guo, W. Shen, M. Li, R. He, *Appl. Catal. B-Environ.* **2020**, *263*, 118324.

[94] H. Zhang, Y. Lv, C. Chen, C. Lv, X. Wu, J. Guo, D. Jia, *Appl. Catal. B-Environ.* **2021**, *298*, 120611.

[95] H. Sun, J.-M. Yang, J.-G. Li, Z. Li, X. Ao, Y.-Z. Liu, Y. Zhang, Y. Li, C. Wang, J. Tang, *Appl. Catal. B-Environ.* **2020**, *272*, 118988.

[96] S. Li, C. Xi, Y.-Z. Jin, D. Wu, J.-Q. Wang, T. Liu, H.-B. Wang, C.-K. Dong, H. Liu, S. A. Kulinich, *ACS Energy Lett.* **2019**, *4*, 1823.

[97] X. Wang, H. Huang, J. Qian, Y. Li, K. Shen, *Appl. Catal. B-Environ.* **2023**, *325*, 122295.

[98] Y. Wu, X. Tao, Y. Qing, H. Xu, F. Yang, S. Luo, C. Tian, M. Liu, X. Lu, *Adv. Mater.* **2019**, *31*, 1900178.

[99] H. Xu, T. Liu, S. Bai, L. Li, Y. Zhu, J. Wang, S. Yang, Y. Li, Q. Shao, X. Huang, *Nano Lett.* **2020**, *20*, 5482.

[100] D. Chen, R. Lu, Z. Pu, J. Zhu, H.-W. Li, F. Liu, S. Hu, X. Luo, J. Wu, Y. Zhao, *Appl. Catal. B-Environ.* **2020**, *279*, 119396.

[101] H. Sun, Y. Min, W. Yang, Y. Lian, L. Lin, K. Feng, Z. Deng, M. Chen, J. Zhong, L. Xu,

ACS Catal. **2019**, *9*, 8882.

[102] X. Zheng, P. Cui, Y. Qian, G. Zhao, X. Zheng, X. Xu, Z. Cheng, Y. Liu, S. X. Dou, W. Sun, *Angew. Chem. Int. Ed.* **2020**, *59*, 14533.

[103] L. Yang, R. Liu, L. Jiao, *Adv. Funct. Mater.* **2020**, *30*, 1909618.

[104] L. Wu, L. Yu, F. Zhang, B. McElhenny, D. Luo, A. Karim, S. Chen, Z. Ren, *Adv. Funct. Mater.* **2021**, *31*, 2006484.

[105] T. Li, S. Li, Q. Liu, J. Yin, D. Sun, M. Zhang, L. Xu, Y. Tang, Y. Zhang, *Adv. Sci.* **2020**, *7*, 1902371.

[106] L. Chai, Z. Hu, X. Wang, Y. Xu, L. Zhang, T. T. Li, Y. Hu, J. Qian, S. Huang, *Adv. Sci.* **2020**, *7*, 1903195.

[107] Y. Pan, K. Sun, S. Liu, X. Cao, K. Wu, W.-C. Cheong, Z. Chen, Y. Wang, Y. Li, Y. Liu, *J. Am. Chem. Soc.* **2018**, *140*, 2610.

[108] M. R. Liu, Q. L. Hong, Q. H. Li, Y. Du, H. X. Zhang, S. Chen, T. Zhou, J. Zhang, *Adv. Funct. Mater.* **2018**, *28*, 1801136.
